# Full-dimensional quantum stereodynamics of the non-adiabatic quenching of OH(A^2^Σ^+^) by H_2_

**DOI:** 10.1038/s41557-021-00730-1

**Published:** 2021-08-09

**Authors:** Bin Zhao, Shanyu Han, Christopher L. Malbon, Uwe Manthe, David. R. Yarkony, Hua Guo

**Affiliations:** 1 Department of Chemistry and Chemical Biology, University of New Mexico, Albuquerque, NM USA; 2 Theoretische Chemie, Fakultät für Chemie, Universität Bielefeld, Bielefeld, Germany; 3 Department of Chemistry, Johns Hopkins University, Baltimore, MD USA

**Keywords:** Reaction kinetics and dynamics, Excited states

## Abstract

The Born–Oppenheimer approximation, assuming separable nuclear and electronic motion, is widely adopted for characterizing chemical reactions in a single electronic state. However, the breakdown of the Born–Oppenheimer approximation is omnipresent in chemistry, and a detailed understanding of the non-adiabatic dynamics is still incomplete. Here we investigate the non-adiabatic quenching of electronically excited OH(A^2^Σ^+^) molecules by H_2_ molecules using full-dimensional quantum dynamics calculations for zero total nuclear angular momentum using a high-quality diabatic-potential-energy matrix. Good agreement with experimental observations is found for the OH(X^2^Π) ro-vibrational distribution, and the non-adiabatic dynamics are shown to be controlled by stereodynamics, namely the relative orientation of the two reactants. The uncovering of a major (in)elastic channel, neglected in a previous analysis but confirmed by a recent experiment, resolves a long-standing experiment–theory disagreement concerning the branching ratio of the two electronic quenching channels.

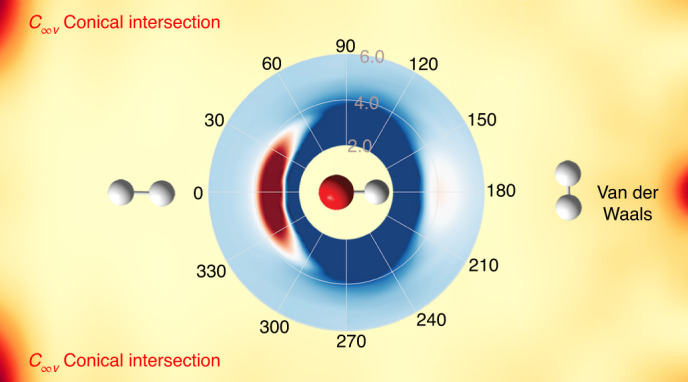

## Abstract (web summary)

The breakdown of the Born–Oppenheimer approximation is omnipresent in chemistry and detailed understanding of non-adiabatic dynamics is still incomplete. Now, the non-adiabatic quenching of electronically excited OH(A^2^Σ^+^) molecules by H_2_ has been investigated using full-dimensional quantum dynamics calculations and a high-quality diabatic-potential-energy matrix, providing insight into the branching ratio of the two electronic quenching channels.

## Main

Although the Born–Oppenheimer approximation^1^, which assumes separability of nuclear and electronic motion, is widely accepted for characterizing reactions in their ground electronic states, there is general agreement that dynamics can be impacted by excited electronic states near an electronic degeneracy, such as conical intersections (CI), where the electronic and nuclear coordinates are strongly coupled. While ultrafast non-adiabatic transitions near a CI have been extensively studied in photochemistry^2–8^ and non-reactive collisions^9–11^, fewer studies on non-Born–Oppenheimer effects exist for bimolecular reactions^12^. Existing first-principles theories of non-adiabatic reaction dynamics mostly deal with open-shell atoms, focusing on geometric phase effects^13–15^ or spin–orbit excited electronic states^16–21^. Here we extend the full-dimensional quantum description to the quenching of an electronically excited molecule:

1 $${\mathrm{OH(A}}^{\mathrm{2}}\Sigma{^{\mathrm{ + }}}{\mathrm{) + H}}_{\mathrm{2}} \to {\mathrm{H + H}}_{\mathrm{2}}{\mathrm{O}}\quad \quad \left( {{\mathrm{reactive}}\;{\mathrm{quenching}}} \right)$$

2 $$\to {\mathrm{OH(X}}^{\mathrm{2}}{\Pi}{\mathrm{) H}}_{\mathrm{2}}\quad \quad \left( {{\mathrm{non - reactive}}\;{\mathrm{quenching}}} \right)$$

3 $$\to {\mathrm{OH(A}}^{\mathrm{2}}\Sigma{^{\mathrm{ + }}}{\mathrm{) + H}}_{\mathrm{2}}\quad \quad \left( {{\mathrm{elastic}}\;{\mathrm{and}}\;{\mathrm{inelastic}}\;{\mathrm{scattering}}} \right)$$

As both non-radiative quenching channels (channels 1 and 2) necessarily require transitions between electronic states via CIs^22,23^, this system offers a prototype for fundamentally understanding non-adiabatic dynamics in bimolecular collisions. It is also of great practical relevance to the laser-induced fluorescence monitoring of the omnipresent OH radicals in atmospheric chemistry and in combustion^24,25^.

Pioneering experiments by Lester and co-workers identified the reactive quenching channel 1 (ref. ^26^) and found a bimodal kinetic energy distribution of the H co-product, suggesting complex dynamics with at least two reaction pathways^27–29^. These intrabeam measurements were confirmed by a crossed-beam experiment by Ortiz-Suárez et al.^30^. Later experiments by the Lester group investigated the non-reactive quenching channel 2 with quantum state resolution^28,31–33^. The OH(X^2^Π) product was found to be vibrationally cold but rotationally hot, with a propensity for the A′ component of the Λ-doublet. Furthermore, the Lester group reported that the branching ratio between the reactive and non-reactive quenching channels favours the former^28^. However, more recently, Brouard and co-workers determined the cross sections of the inelastic channel and the total (reactive plus non-reactive) quenching in absolute units, finding that the inelastic channel was three times larger than that of the total quenching channel^34^. The existence of quantum state-resolved experimental data makes this system a fertile proving ground for theoretical investigations.

**Fig. 1:**
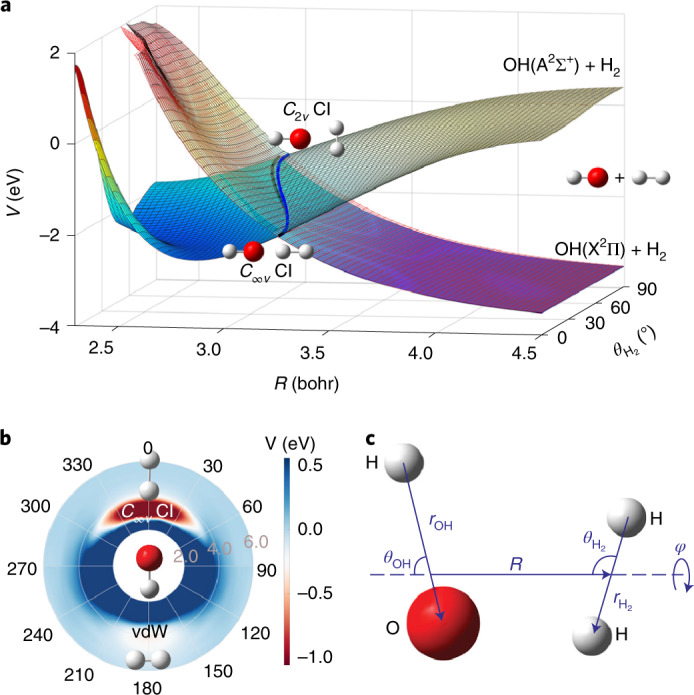
Two-dimensional plots of the interaction PESs. **a**, Two-dimensional cuts of the three adiabatic PESs in the *R* and $\theta _{H_2}$ coordinates in the H_2_–OH orientation. The reference geometry is chosen at the *C*_∞__*v*_ MEX. The blue and black thick lines represent the 2^2^A–3^2^A and 1^2^A–2^2^A CI seams, respectively. **b**, Polar plot of the adiabatic 3^2^A surface in the entrance channel with *R* and $\theta _{\mathrm{OH}}$ as the radius (in units of bohr) and angle (in units of degree), respectively, with the remaining coordinates relaxed. **c**, The coordinates used in these plots and quantum calculations are defined. The plots of PESs show the anisotropy that determines the stereodynamics of the non-adiabatic quenching of OH(A) by H_2_.

Early ab initio calculations identified a T-shaped (*C*_2__*v*_) CI with OH pointing its O end to H_2_ (ref. ^35^), which was hypothesized to be responsible for efficient quenching of OH(A^2^Σ^+^). Subsequent studies by Yarkony and Hoffman revealed that this CI seam actually spans the entire planar (*C*_*s*_) geometry, extending from *C*_2__*v*_ to *C*_∞__*v*_ geometries^36,37^. In Fig. 1a, this confluence of the CI seam is shown as a function of the H_2_–OH distance and the H_2_ rotational angle, with O always pointing to H_2_. Further studies by Dillon and Yarkony explored the non-planar portion of the configuration space and identified additional regions of this CI seam, which facilitate a non-planar insertion pathway of HO into H_2_ (ref. ^38,39^), and lead eventually to the H + H_2_O channel (channel 1). More recently, several multidimensional potential energy surfaces (PESs) have been reported^32,40–44^. Specifically, reduced- and full-dimensional diabatic potential matrices (DPEMs) with two^32,41^, three^42,43^ and four^44^ electronic states have been developed. The latest four-state global DPEM offers the highest fidelity in reproducing ab initio energies and couplings^44^.

These high-quality DPEMs have opened the door for dynamical studies^40–42,45^. While the non-adiabatic dynamics can only be accurately characterized quantum mechanically, such calculations are challenging because of the large energy release (>4 eV), large accessible phase space and complex multistate dynamics^12,40^. So far, detailed quantum dynamics calculations have been restricted to planar geometries with two electronic states^41,45^. However, this modelling is insufficient since it neglects important non-planar non-adiabatic dynamics. On the other hand, full-dimensional trajectory surface hopping (TSH), and some preliminary quantum mechanical studies, have been employed to gain insights into the quenching events^42^. Interestingly, the results favoured the non-reactive quenching channel, opposite to the original analysis^28^. Since the reactive/non-reactive quenching branching ratio is of fundamental importance in this non-adiabatic process, a definitive re-evaluation is necessary.

We report here a detailed full-dimensional investigation of the non-adiabatic collisional quenching of OH(A^2^Σ^+^) by H_2_ using time-dependent wave packet calculations for zero total nuclear angular momentum (*N*_tot_ = 0) on the recently developed DPEM^44^. We aim to resolve the aforementioned experiment–theory discrepancy, to validate the DPEM by comparing quantum state-resolved product distributions with experiment and to gain insight into the stereodynamics of this prototypical non-adiabatic process.

## Results

As shown in Table 1, the calculated fraction for the non-reactive quenching channel (*f*_2_) at the collision energy of 0.05 eV is 0.123, and that for reactive quenching (*f*_1_) is 0.098. The former is in good agreement with the experimental value (0.12(5)) (ref. ^28^). However, our results suggest that non-reactive quenching is slightly favoured over reactive quenching at this energy, and this preference increases with increasing collision energy. Although the minor insertion pathway is ignored in our calculations, its effect on the branching ratio is considered unimportant. This preference, consistent with the earlier theoretical results based on a DPEM of Collins et al.^42^, is in sharp contrast to the experimental report by Dempsey et al.^28^, which showed a dominant reactive quenching channel. As discussed in the next section, we attribute the experiment–theory discrepancy to the large yield of the adiabatic elastic and inelastic channel (channel 3), which was neglected in the previous analysis of the branching ratio^28^. To this respect, the recent work of Brouard et al.^34^ indicated that the inelastic scattering cross section is three times as large as the quenching one (channel 1 + channel 2) for the ground rotational state of the OH(A) reactant (*N*_OH_ = 0), confirming our results.

**Table 1:** Branching fractions for the three channels of OH(A^2^Σ^+^) + H_2_ at three different collision energies with zero total nuclear angular momentum (*N*_tot_ = 0)

*E*_c_ (eV)		H + H_2_O	H_2_ + OH(X^2^Π)			H_2 _+ OH(A^2^Σ^+^)
			Total	1A′	1A″	
0.05	Exp.		0.12(5)			
	*N*_tot_ = 0 [*b* = 0]^a^	0.098 [0.075]	0.123 [0.293]	0.086	0.037	0.772 [0.632]
	[All *b*]	[0.009]	[0.055]			[0.930]
0.16	*N*_tot_ = 0 [*b* = 0]	0.111 [0.090]	0.282 [0.343]	0.186	0.096	0.606 [0.568]
0.30	*N*_tot _= 0 [*b* = 0]	0.187 [0.101]	0.339 [0.372]	0.219	0.119	0.476 [0.527]

^a^The values in square brackets were obtained from TSH calculations, which include zero impact parameter *b* = 0 and all relevant *b* values.

**Fig. 2:**
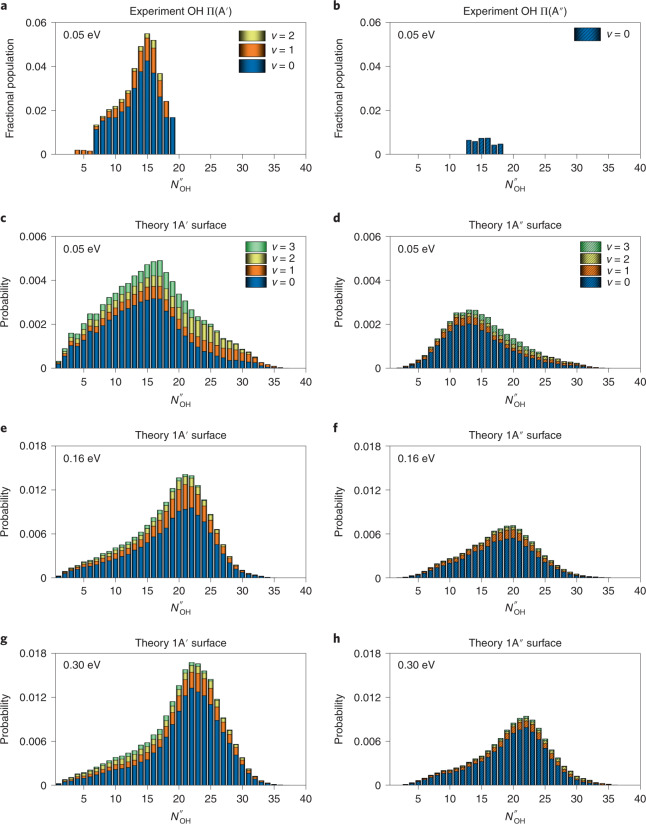
OH(X^2^Π) ro-vibrational state-resolved probabilities in the non-reactive quenching channel. **a**,**b**, Experimental^31^ results at the collision energy of 0.05 eV. **c**–**h**, Theoretical results at three collision energies: 0.05 eV (**c**,**d**); 0.16 eV (**e**,**f**); and 0.30 eV (**g**,**h**). The experimental results in the left and right columns are for the F_1_ (Ω = 3/2) spin–orbit manifold of OH Π(A′) (**a**) and OH Π(A″) (**b**), respectively, where Ω is the projections total electronic (spin and orbital) angular momenta onto the internuclear axis; the theoretical results in the left and right columns are for the 1A′ (**c**,**e**,**g**) and 1A″ (**d**,**f**,**h**) states, respectively. The theoretical results show a good agreement with experimental observations, which show that the OH(X) products are dominantly in the ground vibrational state (*v* = 0) with a broad rotational state distribution peaking at *N*″_OH_ = 15. The weak vibrational excitation stems from the fact that the O–H bond length at the *C*_∞__*v*_ MEX is similar to the equilibrium values of OH(A^2^Σ^+^) and OH(X^2^Π), and the rotational excitation is related to anisotropy on the 1^2^A and 2^2^A state surfaces. Source data

The internal state distributions of the OH(X^2^Π) product were calculated at three collision energies. Figure 2 displays the OH(X^2^Π) ro-vibrational state-resolved probabilities on both the 1A′ and 1A″ PESs. It should be noted that they are related, but do not directly correspond, to the populations of the Π(A′)- and Π(A″)-doublet components of the OH(X^2^Π) product^46^. The present calculations show that the OH(X) products are dominantly in the ground vibrational state with a broad rotational state distribution peaking at *N*_OH_ = 17, in good agreement with experimental observations^31^, shown in the top panels of the same figure. The peak of the distribution shifts towards higher *N*_OH_ with increasing collision energy. Furthermore, Dempsey and co-workers reported that the OH(X^2^Π) products are mainly formed in the Π(A′) component of this degenerate electronic state^31^, a trend supported by our calculated distributions.

**Fig. 3:**
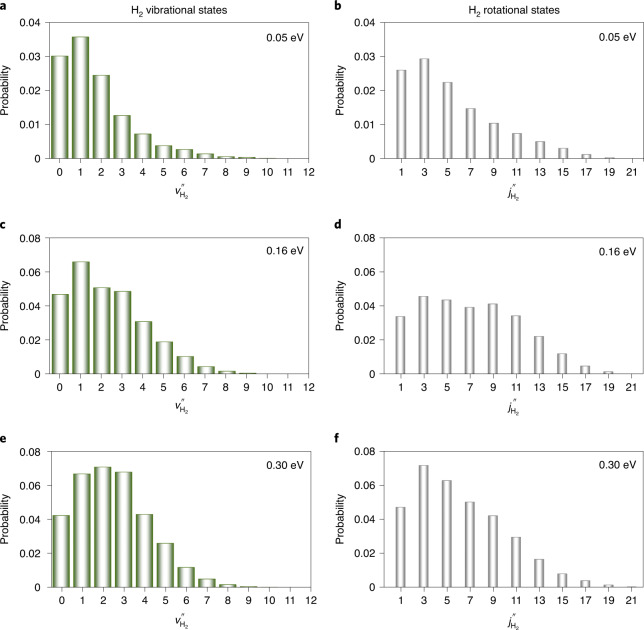
Calculated H_2_ vibrational and rotational state-resolved probabilities in the non-reactive quenching channel. **a**–**f**, The vibrational (**a**,**c**,**e**) and rotational (**b**,**d**,**f**) states of H_2_ at three collision energies: 0.05 eV (**a**,**b**); 0.16 eV (**c**,**d**); and 0.30 eV (**e**,**f**). The H_2_ products are both vibrationally and rotationally hot. The vibrational excitation is attributed to the stretched H–H bond at the *C*_∞__*v*_ MEX, and the rotational excitation is related to anisotropy on the 1^2^A and 2^2^A state surfaces. Source data

The H_2_ ro-vibrational state distribution associated with OH(X^2^Π) has also been calculated, although there is currently no experimental data with which to compare it. In Fig. 3, the final H_2_ vibrational and rotational state-resolved probabilities are displayed. The H_2_ products are both vibrationally and rotationally hot, and only odd H_2_ rotational states are formed. The absence of the even H_2_ rotational states results from the permutation symmetry of the DPEM within our simplified Hamiltonian, as discussed in Supplementary Information. The H_2_ vibrational state distribution extends to higher vibrational states when the collision energy is increased.

## Discussion

First, it is necessary to provide some details regarding the electronic states and potential pathways that are involved in the OH(A^2^Σ^+^) + H_2_ reaction. In the entrance channel, the 1^2^A(A″)/2^2^A(A′) and 3^2^A(A′) states correlate to the doubly degenerate OH(X^2^Π) + H_2_ and the OH(A^2^Σ^+^) + H_2_ asymptotes, respectively. The A′ and A″ labels refer to planar geometries that show *C*_*s*_ symmetry. In the reactive quenching channel, the 1^2^A state correlates adiabatically with the H_2_O + H products. There are two CI seams, as marked in Fig. 1a (and shown in detail in Supplementary Video 2). The first one connects the 2^2^A and 3^2^A states, extending from the *C*_2__*v*_ to *C*_∞__*v*_ geometries and with *C*_*s*_ geometries in between^36,37^. Here the 2^2^A/3^2^A states transform according to irreducible representations of B_2_/A_1_, Π/Σ and A′/A′, respectively. The second CI seam connects the 1^2^A and 2^2^A states^38,39^. Two non-adiabatic quenching pathways for channel 1 are possible: a direct abstraction pathway with two successive transitions near the 2^2^A–3^2^A and 1^2^A–2^2^A seams in the valence region, and an insertion pathway where OH is inserted into the H_2_ bonds in the 2^2^A or 1^2^A state after an initial transition from the 3^2^A state. The insertion pathway can access the *C*_3__*v*_ and *D*_3__*h*_ structures, where the energy in the three O–H bonds may be randomly redistributed. Both pathways can lead to reactive and non-reactive quenching, and might be responsible for the bimodality in channel 1 and the population of the A′ and A″ Λ-doublet states of the OH(X^2^Π) product.

Our wave packet calculations revealed that the fate of the collision between OH(A^2^Σ^+^) and H_2_ is strongly controlled by stereodynamics, which has important consequences for the quenching process. As recognized before^35^, the OH(A^2^Σ^+^) + H_2_ entrance channel features a barrierless access to a T-shaped van der Waals (vdW) well with the H end of OH pointing towards H_2_ ($\theta _{\mathrm{H}_{2}} = 90^\circ$ and *θ*_OH_ = 180°), which has a depth of 0.34 eV. The spectrum and lifetimes of predissociative states in this well have been a subject of extensive studies by Lester and co-workers^35^. However, along this path there is no easy access to the 2^2^A–3^2^A CI seam because of a large barrier separating the vdW well from the upper cone of the CI, which corresponds to *θ*_OH_ changing from 180° to 0°, as illustrated in Fig. 1b and in more detail in Supplementary Fig. 1. As a result, the wave packet entering the vdW well is largely reflected back to the OH(A^2^Σ^+^) + H_2_ channel by the repulsive wall. Noting that the O–H and H–H bond lengths in the vdW well (1.975 bohr and 1.461 bohr, respectively) are essentially the same as those of the free molecules, there is thus little vibrational excitation. The T-shape also dictates that rotational excitation is minimal because little torque results from momentum transfer. These features are shown in Supplementary Fig. 2. Importantly, this particular steric approach has a large cone of acceptance leading to the large yield for channel 3 shown in Table 1, although the dominance of this elastic and inelastic channel is weakened at higher energies. The adiabatic elastic and inelastic channel has been shown by Brouard et al. to be quite dominant^34^.

**Fig. 4:**
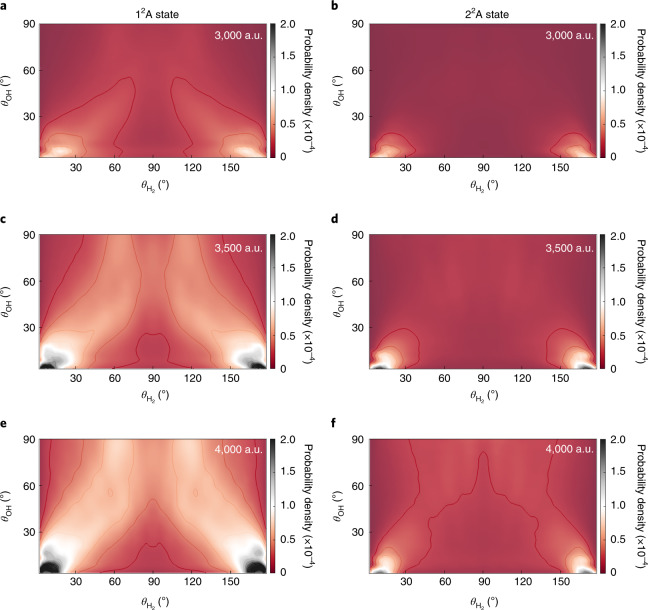
Plots of the probability densities of the wave packet after non-adiabatic quenching in the $\theta _{H_2}$ and *θ*_OH_ coordinates. **a**–**f**, The left and right columns are for the wave packet on the 1^2^A (**a**,**c**,**e**) and 2^2^A (**b**,**d**,**f**) state surfaces, respectively, at three propagation times: 72.6 fs (**a**,**b**); 84.7 fs (**c**,**d**); and 96.8 fs (**e**,**f**). The probability densities were obtained by integrating over the remaining four coordinates. The wave packets emerging on the 1^2^A and 2^2^A state surfaces are largely located near the H–H–O–H collinear geometry ($\theta _{\mathrm{H}_{2}} = 0^\circ ,180^\circ$ and *θ*_OH_ = 0°), indicating the dominant contribution of the *C*_∞__*v*_ MEX. Source data

On the other hand, the upper cone of the 2^2^A–3^2^A CI can be accessed when OH(A^2^Σ^+^) approaches H_2_ with its O end (*θ*_OH_ = 0°) also without a barrier as shown in Fig. 1a,b. This CI seam has been identified in previous work^36,37^, but most discussion on its effect has focused on the seam in *C*_2__*v*_ symmetry^32,35^. Interestingly, our quantum dynamics calculation indicates that the wave packets emerging on the 1^2^A and 2^2^A states are largely located near the H–H–O–H collinear geometry ($\theta _{\mathrm{H}_{2}}{\mathrm{ = 0}}^\circ {\mathrm{,180}}^\circ$ and *θ*_OH_ = 0°), as shown in Fig. 4 (and Supplementary Video 1 and Supplementary Fig. 6). This observation underscores the dominance of the collinear reaction pathway over the *C*_2__*v*_ pathway proposed in previous work^32,35^. A closer examination of the CI seam reveals the origin of this behaviour: the asymptote 3^2^A PES has a large anisotropy with respect to the H_2_ rotational angle ($\theta _{\mathrm{H}_{2}}$), as shown in Fig. 1a (and Supplementary Fig. 7), which guides the incoming wave packet to the linear geometry. Indeed, the minimum energy crossing (MEX) with *C*_∞__*v*_ symmetry is ~0.631 eV lower than that at *C*_2__*v*_ symmetry, the *C*_*s*_ symmetry seam linking both structures, as shown in Fig. 1a (and Supplementary Table 1).

Steric effects, controlled by PES anisotropy, have long been recognized in adiabatic collisions^47–49^. In an activated reaction, the access of the reactive transition state is often facilitated by a cone of acceptance^50^, which can be controlled by reactant orientation. Stereodynamics may also impact product state distributions^51^ and sometimes product branching^52^. The influence of steric effects on non-adiabatic dynamics is expected, although such examples in collision processes are few and far between^53,54^. The non-adiabatic quenching process discussed here serves as an excellent example of stereodynamics in non-adiabatic barrierless scattering between molecular reactants.

On the basis of the stereodynamics described above, both the experimental and theoretical results can be rationalized. To begin with, we reconcile the aforementioned controversy concerning the branching ratio between the two quenching channels. In the work of Dempsey et al.^28^, the yield of the non-reactive quenching channel (*f*_2_) was measured directly; however, the yield for the reactive quenching channel (*f*_1_) was derived with the assumption that the OH(A^2^Σ^+^) radiative quenching is near completion, because of the fast fluorescence decay time of OH(A^2^Σ^+^) (165 ns) and the corresponding small yield^32^. Unfortunately, the existence of this elastic and inelastic channel was not considered in their branching ratio model. The recent experiment of Brouard et al.^34^ found that quenching (reactive and non-reactive) represents only a minority of the scattering outcome, with a cross section that is only one-third of that for inelastic scattering. When elastic scattering is included, the adiabatic elastic and inelastic channel would have an even larger cross section. The experiment of Brouard et al.^34^ was performed under thermal conditions (~0.039 eV), which is close to the collision energy of 0.05 eV. Indeed, if we subtract the calculated fraction of channel 3 (*f*_3_ = 0.772) from the experimental value for channels 1 and 3 (0.875), the fraction of channel 1 (*f*_1_) would be 0.103, which is very close to our calculated value of 0.098. On the basis of both the latest experiment and our theoretical calculations, we propose a reinterpretation of the earlier experimental results by including the elastic and inelastic yield, which leads to the conclusion that the reactive quenching is no longer the dominant channel. This conclusion is consistent with the earlier theoretical results of Collins et al.^42^ using a different DPEM.

We note in passing that the dominance of elastic and inelastic scattering on the upper electronic state over quenching was also observed in the non-reactive collisions of OH(A^2^Σ^+^) with heavy rare gas (RG) atoms (Kr^10^ and Xe^11^). The non-adiabatic transitions are also sterically selective: the RG–H–O vdW well on the upper electronic state is separated from the RG–O–H CI, which couples to the lower electronic states^9–11^. The OH(X^2^Π) product was also found to be rotationally hot but vibrationally cold^9^. Despite notably different kinematics, these two systems seem to share many similarities in collision dynamics.

An important caveat concerning the aforementioned branching ratio obtained from our quantum calculations is the lack of contributions of higher partial waves. While *N*_tot_ > 0 quantum scattering calculations are beyond the scope of this work, we have investigated this question using TSH with all accessible impact parameters (*b*) (*b*_max_ = 5.5 Å or *N*^max^_tot_ = 35). The calculated fractions for the three channels at *E*_c_ = 0.05 eV are as follows: 0.009 (channel 1), 0.055 (channel 2) and 0.93 (channel 3), shown in Table 1. The increased dominance of the elastic and inelastic channel at large impact parameters is readily understood as the H_2_–OH approach to the CI region being partially blocked by a centrifugal barrier while the elastic and inelastic channel is hardly affected. The inclusion of the higher partial waves in the entrance channel does not qualitatively change the conclusion.

**Fig. 5:**
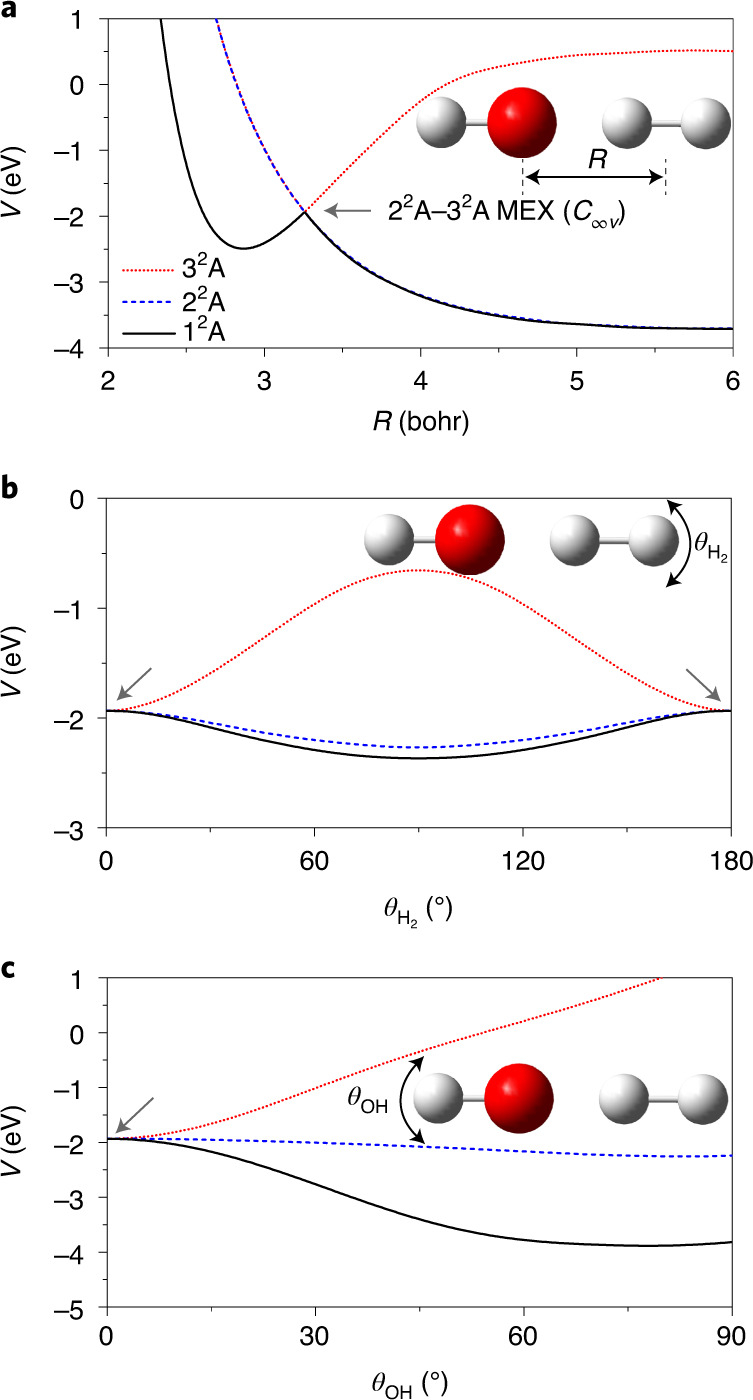
One-dimensional cuts of the three adiabatic surfaces in the H_2_–OH orientation. **a**, Dependence on the *R* coordinate. **b**, Dependence on the $\theta _{\mathrm{H}_{2}}$ angle. **c**, Dependence on the $\theta _{\mathrm{OH}}$ angle. The reference geometry is taken at the *C*_∞__*v*_ MEX (Supplementary Table 1). The OH and H_2_ rotational excitations are attributed to the anisotropy on the 1^2^A and 2^2^A state PESs.

Further information is provided for the product state distribution in the non-reactive quenching channel. The OH rotational excitation can be attributed to the large anisotropy on the 1^2^A and 2^2^A state PESs, which exerts a strong torque on the departing OH fragment. This driving force exists not only in *C*_2__*v*_, discussed in previous work^32^ (and shown in Supplementary Fig. 8) but also in *C*_∞__*v*_. This is illustrated in Fig. 5 where the potential along the *θ*_OH_ coordinate is displayed at the collinear CI seam. The A′ PES has larger anisotropy than the A″ PES. On the other hand, the anisotropy along the $\theta_{{\mathrm{H}}_2}$ angle is relatively small, leading to less rotational excitation of the H_2_ product. We note that the agreement with experiment is not quantitative, which could be due to inaccuracies in the DPEM and/or the lack of higher partial waves and/or incomplete experimental data. The weak OH vibrational excitation stems from the fact that the equilibrium bond lengths of OH(A^2^Σ^+^) (1.901 bohr) and OH(X^2^Π) (1.825 bohr) are similar. They are also quite close to that at the *C*_∞__*v*_ MEX (1.820 bohr). On the other hand, the H_2_ vibrational excitation can be attributed to the stretched H–H bond at the *C*_∞__*v*_ MEX. The H–H distance at the MEX(*C*_∞__*v*_) is 1.776 bohr, which is notably larger than its equilibrium value (1.406 bohr) (see Supplementary Table 1 for further details). As shown in Supplementary Video 1, the wave functions on the 1^2^A and 2^2^A surfaces first shrink to a small H–H distance right after the non-adiabatic transitions and bounce back from the potential wall at the small H–H value. Subsequently, the wave packet moves in a zigzag path in the exit channel, indicating vibrational excitation. Finally, the notable population of OH(X) states on the A″ PES underscores the importance of out-of-plane dynamics and the CI seam between the two lowest electronic states leading to the OH(X^2^Π) + H_2_ products.

## Conclusions

The collisional quenching of OH(A^2^Σ^+^) by H_2_ has served as an important prototype for understanding non-adiabatic dynamics in bimolecular collisions. Although the underlying CIs have been identified for some time, a detailed quantum characterization of the non-adiabatic dynamics has not been achieved until now. Using a highly accurate diabatic-potential-energy matrix that includes four lowest-lying electronic states, we report here a detailed full-dimensional quantum dynamics study of this prototypical non-adiabatic process involving four atoms and six coordinates. Our results revealed that the fate of the OH(A^2^Σ^+^) + H_2_ collision is largely determined by stereodynamics, namely the relative orientation between the two collisional partners. The quenching is made possible with the H_2_–OH approach as H_2_–HO collisions are ineffective in accessing the CI seam. Furthermore, non-adiabatic transitions in the former orientation occur mostly near the collinear *C*_∞__*v*_ CI seam, rather than the *C*_2__*v*_ CI seam proposed in previous work^32,35^. Most interestingly, the existence of a major elastic and inelastic channel, not included in the original analysis of the branching ratio of the two quenching channels^28^, suggests a reinterpretation of the earlier experimental results^28^ that is consistent with our theoretical findings and the new experiment^34^. This resolves a long-standing experiment–theory discrepancy concerning the reactive/non-reactive branching ratio. Finally, the OH(X^2^Π) and H_2_ product state distributions were calculated and compared well with available experimental results. These results validate the accuracy of the recently developed diabatic-potential-energy matrix and shed valuable light on the complex non-adiabatic dynamics involved in the OH(A^2^Σ^+^) quenching.

## Methods

### Quantum wave packet method

#### Hamiltonian and basis representation

We employed a full-dimensional quantum wave packet method to study the collisional quenching of OH(A^2^Σ^+^) by molecular hydrogen on the recently constructed four-state DPEM^44^. The calculations were restricted to zero total nuclear angular momentum (*N*_tot_ = 0) and the electronic (spin and orbital) angular momenta were ignored. These restrictions substantially reduce the numerical costs and render the calculations feasible. Since the electronic angular momenta are relatively small compared with the large rotational excitation in the OH(X) and H_2_ products after non-reactive quenching, it is reasonable to ignore their contributions in the total angular momentum so that *N*_tot_ is considered as a good quantum number. We note in passing that the Renner–Teller coupling between the two lower adiabatic states near linearity is zero for *N*_tot_ = 0.

The Hamiltonian and discretization for diatom–diatom systems have been defined in detail before, for example, in ref. ^55^. Here only a brief description is given. As shown in Fig. 1c, the H_2_ + OH Jacobi coordinates are denoted as $(R,r_{\mathrm{H}_{2}},r_{\mathrm{OH}},\theta _{\mathrm{H}_{2}},\theta _{\mathrm{OH}},\varphi )$. The Hamiltonian in the diabatic representation reads:

4 $$\hat H = \hat T{\mathbf{I}}_4 + \left( {\begin{array}{*{20}{c}} {V_{11}} & {V_{12}} & {V_{13}} & {V_{14}} \\ {V_{21}} & {V_{22}} & {V_{23}} & {V_{24}} \\ {V_{31}} & {V_{32}} & {V_{33}} & {V_{34}} \\ {V_{41}} & {V_{42}} & {V_{43}} & {V_{44}} \end{array}} \right),$$

where **I**_4_ is a 4×4 identity matrix, and the elements of the symmetric diabatic-potential-energy matrix ${\hat{\mathbf V}}$ depend on the six internal coordinates; that is, $V_{nm}(R,r_{\mathrm{H}_{2}},r_{\mathrm{OH}},\theta _{\mathrm{H}_{2}},\theta _{\mathrm{OH}},\varphi )$, where *n*,*m* = 1,2,3,4. The kinetic energy operator $\hat T$ for *N*_tot_ = 0 ($\hbar$ = 1 hereafter) is

5 $$\begin{array}{ll}\hat T = & - \frac{1}{{2\mu }}\frac{{\partial ^2}}{{\partial R^2}} + \frac{{(\hat N_{tot} - \hat j)^2}}{{2\mu R^2}} - \frac{1}{{2\mu _{\mathrm{H}_{2}}}}\frac{{\partial ^2}}{{\partial r_{\mathrm{H}_{2}}^2}} + \frac{{\hat j_{\mathrm{H}_{2}}^2}}{{2\mu _{\mathrm{H}_{2}}r_{\mathrm{H}_{2}}^2}} \\ & - \frac{1}{{2\mu _{\mathrm{OH}}}}\frac{{\partial ^2}}{{\partial r_{\mathrm{OH}}^2}} + \frac{{\hat j_{\mathrm{OH}}^2}}{{2\mu _{\mathrm{OH}}r_{\mathrm{OH}}^2}},\end{array}$$

where *μ*, $\mu _{{\rm{H}}_2}$ and *μ*_OH_ are the reduced masses for the three radial Jacobi coordinates *R*, $r_{{\mathrm{H}}_{2}}$ and *r*_OH_, respectively; $\hat j_{{\mathrm{H}}_{2}}$ and *ĵ*_OH_ are the rotational angular momentum operators of H_2_ and OH, respectively, and $\hat j = \hat j_{{\mathrm{H}}_{2}} + \hat j_{\mathrm{OH}}$. $\hat N_{\mathrm{tot}}$ is the conserved total nuclear angular momentum operator of the system and restricted to be zero in the present work.

The wave packet is represented in the diabatic representation as

6 $${{\varPsi}}^d = ({{\varPsi}}_1^d,{{\varPsi}}_2^d,{{\varPsi}}_3^d,{{\varPsi}}_4^d)^T,$$

where ${{\varPsi}}_e^d$ is the wave packet component in *e*th diabatic electronic state (the superscript *d* denotes the diabatic representation).

The Hamiltonian and wave functions were represented with the finite basis representation in the diatom–diatom Jacobi coordinates, and the action of the diabatic-potential-energy operator was evaluated on the discrete variable representation (DVR) grid^56^. The sparse transformation between the finite basis representation and DVR allows an efficient propagation of the wave packets^56^. Specifically, the description of the *R* degree of freedom was given by the sine DVR. The $r_{\mathrm{H}_{2}}$ and *r*_OH_ degrees of freedom were described by the potential-optimized DVR, where the corresponding one-dimensional reference potentials were obtained in the reactant asymptotic regions of the ground electronic state surface. Rotational basis functions are constructed by coupling the eigenfunctions of the diatom angular momentum operators in the body-fixed (BF) frame.

#### Initial state, time propagation and analysis

The initial wave packet is prepared in the OH(A^2^Σ^+^) + H_2_ asymptote on the adiabatic PES as a product of a Gaussian wave packet, $G(R)$, and the ro-vibrational states of H_2_ and OH, $\tilde \phi _{\nu _{{\mathrm{H}}_{2}}^0j_{{\mathrm{H}}_{2}}^0}(r_{{\mathrm{H}}_{2}})$ and $\tilde \phi _{\nu _{\mathrm{OH}}^0j_{\mathrm{OH}}^0}(r_{\mathrm{OH}})$, respectively. *G*(*R*) is chosen to be

7 $$G\left( R \right) = \left( {\frac{1}{{\pi {\mathit{\delta}} ^2}}} \right)^{1/4}{\mathrm{exp}}\left[ { - \frac{{\left( {R - R_0} \right)^2}}{{2{\mathit{\delta}} ^2}} - \mathrm{i}k_0R} \right],$$

where *R*_0_, *δ* and *k*_0_ are the central position, width and momentum of the initial Gaussian wave packet, respectively. Before the propagation, the initial wave packet ${{{\varPsi}}}^a = \left( {0,0,{{{\varPsi}}}_3^a,0} \right)^T$ is transformed to the diabatic representation (the superscript *a* denotes the adiabatic representation)

8 $${{\varPsi}}^d = {\mathbf{U}}{{{\varPsi}}}^a,$$

where **U** is the transformation matrix between diabatic and adiabatic representations.

The wave packets were then propagated simultaneously on the four diabatic surfaces using a second-order split-operator scheme^57^. $\hat T$ is independently operated onto each diabatic wave packet, but the operation of ${\hat{\mathbf V}}$ needs to be performed in the adiabatic representation in which the potential energy operator is diagonal. Thus, at each step, the wave packet is first transformed to the adiabatic representation and then transformed back to the diabatic representation after the operation of ${\hat{\mathbf V}}$.

Negative imaginary absorbing potentials were used to prevent wave functions from reaching grid edges. The wave packets on different states may have different kinetic energies along *R* so that an imaginary absorption potential with two segments was used in the *R* coordinate

9 $$\begin{array}{l}V_{\mathrm{abs}}\left( x \right) = \left\{ {\begin{array}{*{20}{c}} { - \mathrm{i}C_1\left( {\frac{{x - x_s^1}}{{x_e^1 - x_s^1}}} \right)^n,x_s^1 \le x \le x_e^1} \\ { - \mathrm{i}C_1 - \mathrm{i}C_2\left( {\frac{{x - x_s^2}}{{x_e^2 - x_s^2}}} \right)^n,x_s^2 \le x \le x_e^2} \end{array}} \right..\\ \end{array}$$

Here the parameters for the first segment in $\left( {x_s^1,x_e^1} \right)$ were selected to remove wave function components with small kinetic energies and the second one in $\left( {x_s^2,x_e^2} \right)$ for the ones with large kinetic energies (that is, the ones after the non-reactive quenching). The absorbing potential in the $r_{\mathrm{H}_{2}}$ coordinate used only one segment. Detailed parameters for the absorbing potentials are listed in Supplementary Table 2.

The total reactive quenching probability, *P*_1_(*E*), was calculated via a flux analysis in the product channel on a dividing surface at $r_{\mathrm{H}_{2}} = r_{\mathrm{flux}}$

10 $$P_{1}\left( E \right) = \frac{1}{{\mu _{\mathrm{H}_{2}}}}\mathop {\sum }\limits_e {\mathrm{Im}}\left. {\left( \left\langle {{{{\varPhi}}}_e^d(E) \left| \frac{\partial }{{\partial r_{\mathrm{H}_{2}}}}{{{{\varPhi}}}}_e^d(E)\right.} \right\rangle \right)} \right|_{r_{\mathrm{H}_{2}} =\, r_{\mathrm{flux}}},$$

where ${{{\varPhi}}}_e^d(E)$ are the diabatic scattering wave functions at the energy *E*, which is calculated by a Fourier transform of the corresponding time-dependent wave packet

11 $${{{\varPhi}}}_e^d\left( E \right) = \frac{1}{{a(E)}}\mathop {\smallint }\limits_0^\infty {\mathrm{d}}t\;{\mathrm{e}}^{{\mathrm{i}}Et}{{\varPsi}}_e^d(t),$$

where *a*(*E*) is the energy component of the initial wave packet. Similarly, a flux analysis in the *R* coordinate was performed on a dividing surface in the asymptotic region (*R* = *R*_flux_). Probabilities for non-reactive quenching and elastic and inelastic scattering were calculated in the adiabatic representation so that the diabatic scattering wave functions ${{{\varPhi}}}_e^d(E)$ need to be transformed to the adiabatic representation, ${{{\varPhi}}}^a = {\mathbf{U}}^T{{{\varPhi}}}^d$. To this end, the elastic and inelastic scattering probability *P*_3_(*E*) is obtained as

12 $$P_{3}\left( E \right) = \frac{1}{\mu }{\mathrm{Im}}\left. {\left( \left\langle {{{{\varPhi}}}_3^a(E)\left|\frac{\partial }{{\partial R}}{{{\varPhi}}}_3^a(E)\right.} \right\rangle \right)} \right|_{R\, =\, R_{\mathrm{flux}}},$$

and the non-reactive quenching probability *P*_2_(*E*) is obtained as

13 $$P_{2}\left( E \right) = \frac{1}{\mu }\mathop {\sum }\limits_{e = 1}^2 {\mathrm{Im}}\left. {\left( \left\langle {{{{\varPhi}}}_e^a(E)\left|\frac{\partial }{{\partial R}}{{{\varPhi}}}_e^a(E)\right.} \right\rangle \right)} \right|_{R\, =\, R_{\mathrm{flux}}},$$

where the terms with *e* = 1 and *e* = 2 correspond to the quenching probabilities for the OH(X^2^Π(A″)) and OH(X^2^Π(A′)) components, respectively.

Final state analysis for the non-reactive quenching was performed on the dividing plane at *R* = *R*_flux_. To resolve the final state information, a projector onto one of the final states, $P_ \bot = \left| {{{{\varPsi}}}_f} \rangle \right.\left. \langle {{{{\varPsi}}}_f} \right|$, was inserted in the bracket in equation (10). A similar procedure was used to extract the final state distributions for the (in)elastic channel.

#### Numerical parameters and limitation

Detailed parameters used in the wave packet calculations are given in Supplementary Table 2. Specifically, four basis functions were used to describe the OH vibration, thus the results for $v_{\mathrm{OH}}^\prime\prime = 3$ might have minor convergence error. The O–H bond is treated as non-reactive, thus the calculations are incapable of considering the insertion mechanism. Despite its limitation, this treatment is valid for the non-reactive quenching channel because the experimental investigation on the collision of OH with D_2_ ruled out the contribution of the insertion mechanism in this channel (no OD products could be observed)^31^.

#### Validation of the DPEM

To validate the DPEM, scattering dynamics on the ground adiabatic state PES obtained from this DPEM was first studied. The reaction probability agrees well with the one obtained on the most accurate ground-state PES of Chen et al.^58^ (Supplementary Fig. 9). This DPEM is further validated by the non-adiabatic dynamics discussed above in comparison with available experimental results.

### TSH method

In addition to the quantum dynamics calculations, we have also carried out TSH calculations in the adiabatic representation using the adiabatic and non-adiabatic trajectory package of Zheng and co-workers^59^. In this case, trajectories corresponding to the ro-vibrational ground states of the OH(A) and H_2_ reactants were sampled using the harmonic oscillator approximation. For all three collisional energies, calculations were performed with the impact parameter *b* fixed to zero; while for 0.05 eV, calculations were also done for all impact parameters with *b*_max_ = 5.5 Å. The trajectories were propagated by solving the Hamilton’s equation in the adiabatic representation. The trajectories are terminated when the separation between the products is larger than 5 Å. Near the CI seam, the trajectory is allowed to make a hop from one electronic state to another, using the fewest switch method of Tully^60^, with the time uncertainty method^61^ combined with stochastic decoherence^62^. For TSH calculations with *b* = 0, about 15,000 trajectories were run at each collisional energy; for calculations including all relevant *b* values, about 50,000 trajectories were run. The branching ratio is determined by *n*_*i*_/*n*_tot_, where *n*_*i*_ is the number of trajectories that end in channel *i* (*i* refers to channels 1, 2 and 3) and *n*_tot_ is the number. The present work did not resolve the Π(A′) and Π(A″) Λ-doublet components of the OH(X) product, but it is, in principle, feasible using the scheme described in ref. ^46^, which has been applied to study the quenching of OH(A) by rare gas atoms^10,11^.

## Online content

Any methods, additional references, Nature Research reporting summaries, source data, extended data, supplementary information, acknowledgements, peer review information; details of author contributions and competing interests; and statements of data and code availability are available at 10.1038/s41557-021-00730-1.

## Supplementary information

Supplementary information Discussion on system symmetry, discussion on Supplementary Videos, Supplementary Tables 1–4 and Figs. 1–4.

Supplementary Video 1 The evolution of the time-dependent adiabatic wave packets on the 1^2^A, 2^2^A and 3^2^A states.

Supplementary Video 2 An animated view of 2D cuts of the adiabatic PESs near the 2^2^A–3^2^A CI seam.

Supplementary Video 3 The evolution of the initial wave packet on the 3^2^A state surface.

## Data Availability

All the data corresponding to the findings of this study are provided in the article and Supplementary Information. Source data are provided with this paper.

## Source data

Source Data Fig. 2 Statistical Source Data in plain text.
Source Data Fig. 3 Statistical Source Data in plain text.
Source Data Fig. 4 Statistical Source Data in plain text.

## Code availability

Fortran code used for the present work is available at GitHub (https://github.com/QDBinZhao/ABR).
