# Supplementary material for: Full-dimensional quantum stereodynamics of the non-adiabatic quenching of OH(A2Σ+) by H2
Source: Nat Chem. 2021 Aug 9;13(9):909–15. doi: 10.1038/s41557-021-00730-1 (PMC8440216; doi:10.1038/s41557-021-00730-1)
Supplement: Supplementary file 1 — Discussion on system symmetry, discussion on Supplementary Videos, Supplementary Tables 1–4 and Figs. 1–4. [file 41557_2021_730_MOESM1_ESM.pdf]

nature portfolio

https://doi.org/10.1038/s41557-021-00730-1

Supplementary information

Full-dimensional quantum stereodynamics of the non-adiabatic quenching of $OH(A^2\Sigma^+)$ by H_2

In the format provided by the authors and unedited

Supplementary Information for

Full-dimensional quantum stereodynamics of the nonadiabatic quenching of $OH(A^2\Sigma^+)$ by H_2

Bin Zhao,[1,2,*] Shanyu Han,[1] Christopher L. Malbon,[3] Uwe Manthe,[2,*] David. R. Yarkony,[3,*] and Hua Guo[1,*]

[1]Department of Chemistry and Chemical Biology, University of New Mexico, Albuquerque, New Mexico, 87131, USA

[2]Theoretische Chemie, Fakultät für Chemie, Universität Bielefeld, Universitätsstraße 25, D-33615 Bielefeld, Germany

[3]Department of Chemistry, Johns Hopkins University, Baltimore, Maryland 21218, USA

Contents

I. Symmetry considerations 2

II. Additional results 3

II.1 Supplementary Videos: nonadiabatic transitions in real-time, animated view of 2D PESs, and evolution of wave packet on the 3^2A state 3

II.2 Supplementary Tables: critical features on the DPEM, parameters of quantum calculations, and symmetry properties of the DPEM. 4

II.3 Supplementary Figures: features of the DPEM, final-state distributions in the (in)elastic channel, the geometry of nonadiabatic transitions in TSH calculation, reaction probability on the adiabatic PES, etc. 7

I. Symmetry considerations

As shown in Figure 3 (b) of the main text, the H_2 products after non-reactive quenching are exclusively populated in odd rotational states. Here we provide some additional explanation. The complete nuclear permutation and inversion (CNPI) group of H_3O is isomorphic to D_{3h}. In the absence of hydrogen exchange with the OH group, the G_4 CNPI subgroup - isomorphic to the C_{2v} point group - is sufficient. In this subgroup, the electronic state correlating to the OH($A^2\Sigma^+$) reactant transforms as A_1, and states correlating to the OH($X^2\Pi$) products transform as B_1 and B_2, as shown in Supplementary Table 3. The character for the A_1 irrep under the H_2 permutation is +1 (even), and that for the B_1 and B_2 irreps are -1 (odd), as shown in Supplementary Table 4. For the initial H_2 rotational state (j_{H_2}=0, even under permutation) starting in the electronically excited state, the total wave functions (electronic + nuclear) is symmetric under permutation, $A_1 \otimes a_1$. Since the electronic wave functions are anti-symmetric after nonadiabatic transitions, the H_2 rotational wave functions must be anti-symmetric to conserve the symmetry of the total wave function. Thus, H_2 rotational states with odd j''_{H_2} are allowed. In the same token, initial odd H_2 rotational states will lead exclusively to even rotational states for the H_2 products.

It should however be noted that the above analysis is based on the neglect of the electronic angular momenta in our model. If we include electronic orbital angular momentum (L_{elec}) and ignore electronic spin, the electronic state correlating to the OH($A^2\Sigma^+$) reactant has L_{elec}=0, and the states correlating to the OH($X^2\Pi$) products have L_{elec}=1. Considering the conservation of total angular momentum (J_{tot}=N_{tot}+L_{elec}), the N_{tot} on the ground electronic states should be opposite to L_{elec}. The explicit inclusion of electronic orbital angular momentum will change the H_2 rotational states by increasing or decreasing j''_{H_2} by one, but it will only have a minor effect on the stereodynamics and the branching ratio because of the small magnitude of electronic angular momenta.

II. Additional results

II.1 Supplementary Videos: nonadiabatic transitions in real-time, animated view of 2D PESs, and evolution of wave packet on the 3^2A state

Supplementary Video 1 depicts the evolution of the time-dependent adiabatic wave packets on the 1^2A, 2^2A, and 3^2A states. The first frame of the video is chosen at a propagation time of 2,000 a.u. instead of the time zero. The two-dimensional (2D) probability densities were obtained by integrating all other four coordinates. As the video shows, the wave packet initially moves on the 3^2A state (panel (c)), and nonadiabatic transitions occur when it reaches the regions of the $C_{\infty v}$ CI seam, where transitions to the 1^2A and 2^2A states occur almost at the same time. On the 1^2A state (panel (a)), the wave packet subsequently branches into the reactive and non-reactive quenching channels; the wave packet on the 2^2A state (panel (b)) can only reach the non-reactive channel. In the 2D plots in H_2 and OH rotational angles, nonadiabatic transitions to the 1^2A and 2^2A states occur preferentially near the co-linear geometry (θ_{H_2}=0°,180° and θ_{OH}=0°).

Supplementary Video 2 provides an animated view of 2D cuts of the adiabatic PESs near the 2^2A-3^2A CI seam. In panel (a), the cut along the R and θ_{H_2} coordinates displays a continuous seam that connects the $C_{\infty v}$ CI to the C_{2v} ones. The $C_{\infty v}$ MEX is the global minimum on that seam. In panel (b), the cut along the R and θ_{OH} coordinates displays a steep slope away from the intersections. The reference geometry is selected as the $C_{\infty v}$ MEX in Supplementary Table 1.

Supplementary Video 3 shows the evolution of the initial wave packet on the 3^2A state surface. Prepared in the ground ro-vibrational states of OH and H_2, The initial wave packet has equal probability density at time zero on the $\theta_{H_2}-\theta_{OH}$ plane (The Gauss-Legendre quadrature weights are removed to better show the steering effect of the 3^2A state surface on the initial wave packet.). The probability density was obtained by integrating all other four coordinates. When OH approaches H_2, the anisotropy of the excited state orient the H_2 and OH pair to three preferable structures: a T-shaped OH-H_2 structure (θ_{H_2}=90° and θ_{OH}=180°) and two linear structures (θ_{H_2}=0°,180° and θ_{OH}=0°). A representative snapshot of Supplementary Video 3 is shown in Supplementary Figure 12. The T-shaped one has the H end of OH pointing to H_2 and corresponds to a van der Walls (vdW) well – with a depth of 0.34 eV. The well starts to attract OH and H_2 into the well even at large OH-H_2 distances (see Supplementary Figure 1). If OH approaches H_2 slowly, most of the wave packet ends up in the well. On the other hand, the part with the O side of OH facing H_2 reaches the region with strong nonadiabatic couplings. When OH-H_2 distance reaches around 5.0 a.u., a significant barrier appears, which separates the approach of the H and O sides of OH to H_2. The wave packet accumulates near the two linear structures in the vicinity of the $C_{\infty v}$ MEXs; Interestingly, no noticeable wave packet is seen near the C_{2v} MEXs. To this end, the picture becomes clear that the wave function always evolves to the local minima on the surface, i.e., the vdW well and the $C_{\infty v}$ MEXs.

II.2 Supplementary Tables: critical features on the DPEM, parameters of quantum calculations, and symmetry properties of the DPEM.

Supplementary Table 1 lists the geometries and energies of some critical features of the DPEM.[1] The geometries of the three MEXs are used as the reference geometries in Figures 1 and 5 of the main text, and in Supplementary Figures 7 and 8.

Supplementary Table 2 provides the parameters used in the wave packet calculations. The long propagation time of 30, 000 a.u. is to converge the quantities at the small collision energy of 0.05 eV, especially for the one in the (in)elastic channel. We point out that only four basis functions are used to describe the OH vibration, thus incapable of considering the insertion mechanism. An early experimental investigation on the collision of OH with D_2 ruled out the contribution of the insertion mechanism in the non-reactive quenching channel.[2]

The symmetry properties of the DPEM elements in Supplementary Table 3 and the character table of the G_4 (C_{2v}) CNPI subgroup in Supplementary Table 4 are used to explain the exclusive population of the *ortho*-H_2 product in the non-reactive quenching channel.

Supplementary Table 1. Geometries and energies of critical features on the DPEM[1] (Energy in eV, radial coordinates in the unit of Bohr, and angular ones in degree).

	E	r_{H_2}	r_{OH}	R	θ_{H_2}	θ_{OH}	φ
2^2A–3^2A ($C_{\infty v}$ MEX)	-1.934	1.776	1.820	3.258	0	0	0
2^2A–3^2A (C_{2v} MEX)	-1.281	1.601	1.841	2.862	90	0	0
1^2A–2^2A (C_{2v} MEX)	-1.326	1.618	1.841	2.832	90	0	0
OH-H_2 vdW (3^2A)	-0.344	1.461	1.975	4.125	90	180	0
OH+H_2 (1^2A)	-4.141	1.406	1.825				
OH+H_2 (3^2A)	0.000	1.402	1.901				

Supplementary Table 2. Parameters used in the quantum calculations (Atomic units are used if not otherwise stated)

<table>
<tr><th colspan="7">Initial wave packet</th></tr>
<tr><td>R_0</td><td colspan="6">11.0</td></tr>
<tr><td>δ</td><td colspan="6">0.35</td></tr>
<tr><td>k_0</td><td colspan="6">$k_0 = \sqrt{2E_0\mu}$ with E_0 =0.15 eV</td></tr>
<tr><th colspan="7">Sizes of basis functions and grids</th></tr>
<tr><td>R</td><td colspan="6">(0.1,16.0), N_1^R =160, N_2^R =458</td></tr>
<tr><td>r_{H_2}</td><td colspan="6">(0.7,5.0), υ_1^{int} =36, υ_1^{asy} =16</td></tr>
<tr><td>r_{OH}</td><td colspan="6">(0.7,5.0), υ_2 =4</td></tr>
<tr><td>j_{H_2}</td><td colspan="6">(0,45)</td></tr>
<tr><td>j_{OH}</td><td colspan="6">(0,40)</td></tr>
<tr><th colspan="7">Absorbing Potential</th></tr>
<tr><td></td><td>n</td><td>C_1</td><td>C_2</td><td>x_s^1</td><td>x_e^2 / x_s^2</td><td>x_e^2</td></tr>
<tr><td>R</td><td>3.0</td><td>0.0025</td><td>0.3</td><td>10.5</td><td>14.5</td><td>16.0</td></tr>
<tr><td>r_{H_2}</td><td>3.0</td><td>3.0</td><td>0.2</td><td>4.0</td><td>5.0</td><td></td></tr>
<tr><td colspan="7">Propagation time/step: 30000.0/10.0</td></tr>
<tr><td colspan="7">r_{H_2} flux plane: 3.2</td></tr>
<tr><td colspan="7">R flux plane: 10.0</td></tr>
</table>

Supplementary Table 3. Symmetry properties of the elements in the diabatic potential energy matrix.[a]

	**D1(A_1)**	**D2(B_2)**	**D3(B_1)**	**D4(A_1)**
**D1(A_1)**	A_1	B_2	B_1	A_1
**D2(B_2)**		A_1	A_2	B_2
**D3(B_1)**			A_1	B_1
**D4(A_1)**				A_1

[a]In the H_2+OH asymptote, the D2 and D3 diabatic states correspond to the A″ and A′ states of the $H_2+OH(X^2\Pi)$ channel, respectively, while the D1 and D4 ones are coupled for the $H_2+OH(A^2\Sigma^+)$ channel.[1]

Supplementary Table 4. Character table of the G_4 (C_{2v}) CNPI subgroup.

**irrep.**	$\boldsymbol{E}$	$\boldsymbol{P_{12}}$	$\boldsymbol{E^*}$	$\boldsymbol{P_{12}^*}$
**A_1**	1	1	1	1
**A_2**	1	1	-1	-1
**B_1**	1	-1	1	-1
**B_2**	1	-1	-1	1

II.3 Supplementary Figures: features of the DPEM, final-state distributions in the (in)elastic channel, the geometry of nonadiabatic transitions in TSH calculation, reaction probability on the adiabatic PES, etc.

In Supplementary Figure 1, one-dimensional plots of the adiabatic 3^2A state PES display the anisotropy that separates the approaches of the OH to H_2 from the H side and from the O side. In panel (a), the vdW well already shows a significant gradient at large *R* values, and it efficiently attracts wave packet to the well. In panel (b), a barrier that separates the O side from the H side of OH is developed during its approach to H_2. The barrier is high enough to prevent the wave packet in the vdW region from accessing the upper cone leading to the CI. If the OH approaches H_2 with the H side, the wave packet enters to the vdW well, leading to (in)elastic scattering on the excited state. If the OH approaches H_2 with the O side, the wave packet reaches the CI and makes nonadiabatic transitions to the 1^2A and 2^2A states.

Supplementary Figure 2 shows the OH(A) and H_2 rotational state-resolved distributions in the (in)elastic channel at three collision energies. No vibrational excitation in either OH(A) or H_2 was found at all the three collision energies. Rotational excitations are minor, but increase at higher collision energies. Due to symmetry, only *para*-H_2 states emerge for initial H2 rotational state of $\hat{j}_{H_2}$ = 0.

Supplementary Figures 3 and 4 show the OH(X) vibrational and rotational state distributions, respectively. They contain the same information as Figure 2 of the main text, but with more detail.

Supplementary Figure 5 shows the comparison of experimental, quantum, and TSH results for the OH(X) vibrational and OH(X, v_{OH}=0) rotational state distributions at the collision energy of 0.05 eV. The experiment in Ref. 9 only measured part of the OH(X) ro-vibrational states, and the vibrational state distribution was obtained from the normalized fits to the experimental state distributions and thus might not be accurate. While the quantum results are limited to N_{tot}=0, the TSH results are obtained for both *b*=0 and all relevant *b* values. Several observations are in order. First of all, the TSH results for *b*=0 and all *b* agree with each other reasonably well, underscoring the minor effect of the centrifugal barrier on the relative state distribution. Secondly, in the left column for the OH(X) vibrational state distribution, all calculations show the dominance of the ground vibrational state, but the quantum results surprisingly predict more vibrational excited OH(X) than the experimental results. The difference might result from the quantum effect at this low collision energy of 0.05 eV (Note that this feature is not observed in the TSH results.). But, this surprising feature could also be a result of an artifact in the current DPEM that was used in our calculations. Both quantum and TSH calculations show a few non-adiabatic transitions at geometries near the van der Walls well structure of the excited state PES (To our understanding, there shouldn't be couplings between the ground and excited electronic states in this region.). At lower collision energy, the OH(A) and H_2 reactants are trapped in the well for a longer time and thus the dynamics are affected more from the artificial transitions. In contrast, at the other two larger collision energies, the population of the vibrationally excited OH(X) are much smaller, as shown in Supplementary Figure 3. Thirdly, in the right column, the TSH results show a colder

rotational state distribution than the quantum ones. Interestingly, the TSH results of Collins et al.[3] for the quenching of OH(A) by D_2 also showed a colder rotational distribution than the experimental results,[2] even though the calculations were done at a higher collision energy. This difference might be due to the semi-classical treatment of the non-adiabatic transitions. Further studies will be needed to provide more insights on the comparison.

Supplementary Figure 6 shows the hopping geometry distribution for the 3^2A-2^2A transitions in the TSH calculations at 0.05 eV. The hopping is clustered near collinear geometries, much like that in Figure 4 of the main text.

Supplementary Figure 7 shows the anisotropy on the 3^2A state PES that exerts a torque on the H_2 to guide it into the $C_{\infty v}$ CI rather than the C_{2v} one.

Supplementary Figure 8 shows 1D cuts of the PESs in the same way as in Figure 5 of the main text except that the reference geometries are chosen at the 2^2A-3^2A(C_{2v}) and 1^2A-2^2A(C_{2v}) MEXs (see Supplementary Table 1). Similar anisotropies on the 1^2A and 2^2A state PESs are apparent along the OH and H_2 rotational angles.

Supplementary Figure 9 shows the reaction probability of the OH(X) + H_2 → H_2O + H reaction on the ground adiabatic state PES obtained from the DPEM. The comparison with the result obtained on the most accurate CXXZ PES[4] is quite reasonable, although the barrier on the DPEM seems to be overestimated slightly. This agreement confirmed the accuracy of the ground state PES provided by the current DPEM and suggested that the excited state PESs should be of comparable accuracy.

Supplementary Figure 10 shows the branching fractions of the reactive quenching, nonreactive quenching, and (in)elastic channels as functions of the collision energy. It should be noted that the (in)elastic channel populations might not be completely converged at low energies due to the long time trapping in the van der Waals well.

In Supplementary Figure 11, populations of the three adiabatic states, defined as the norm of their respective wave packets, are plotted as a function of time. It is clear that following the initial nonadiabatic transitions, the populations of the 1^2A and 2^2A states start to appear. If only planar geometries were considered, the 2^2A(A′) and 3^2A(A′) states would have no coupling with the 1^2A(A″) state. Products of the non-reactive quenching process would thus appear only in the A′ state of OH(X). A CI between these two states leads to both the A′ and A″ components of the OH($X^2\Pi$) product.

Supplementary Figure 12 is a snapshot of Supplementary Video 3 at a propagation time of 4000 a.u. when the wave packet on the 3^2A state PES shows three preferable structures: a T-shaped OH-H_2 vdW structure (θ_{H_2}=90° and θ_{OH}=180°) and two linear structures (θ_{H_2}=0°,180° and θ_{OH}=0°) corresponding to the $C_{\infty v}$ CI.

References:

1. Malbon CL, Zhao B, Guo H, Yarkony DR. On the nonadiabatic collisional quenching of OH(A) by H_2: a four coupled quasi-diabatic state description. *Phys Chem Chem Phys* 2020, **22**(24)**:** 13516-13527.
2. Dempsey LP, Murray C, Cleary PA, Lester MI. Electronic quenching of OH A 2Σ+ radicals in single collision events with H2 and D2: a comprehensive quantum state distribution of the OH X 2Π products. *Phys Chem Chem Phys* 2008, **10**(10)**:** 1424-1432.
3. Collins MA, Godsi O, Liu S, Zhang DH. An ab initio quasi-diabatic potential energy matrix for OH($^2\Sigma$) + H_2. *J Chem Phys* 2011, **135**(23)**:** 234307.
4. Chen J, Xu X, Xu X, Zhang DH. A global potential energy surface for the H_2 + OH ↔ H_2O + H reaction using neural networks. *J Chem Phys* 2013, **138**(15)**:** 154301.

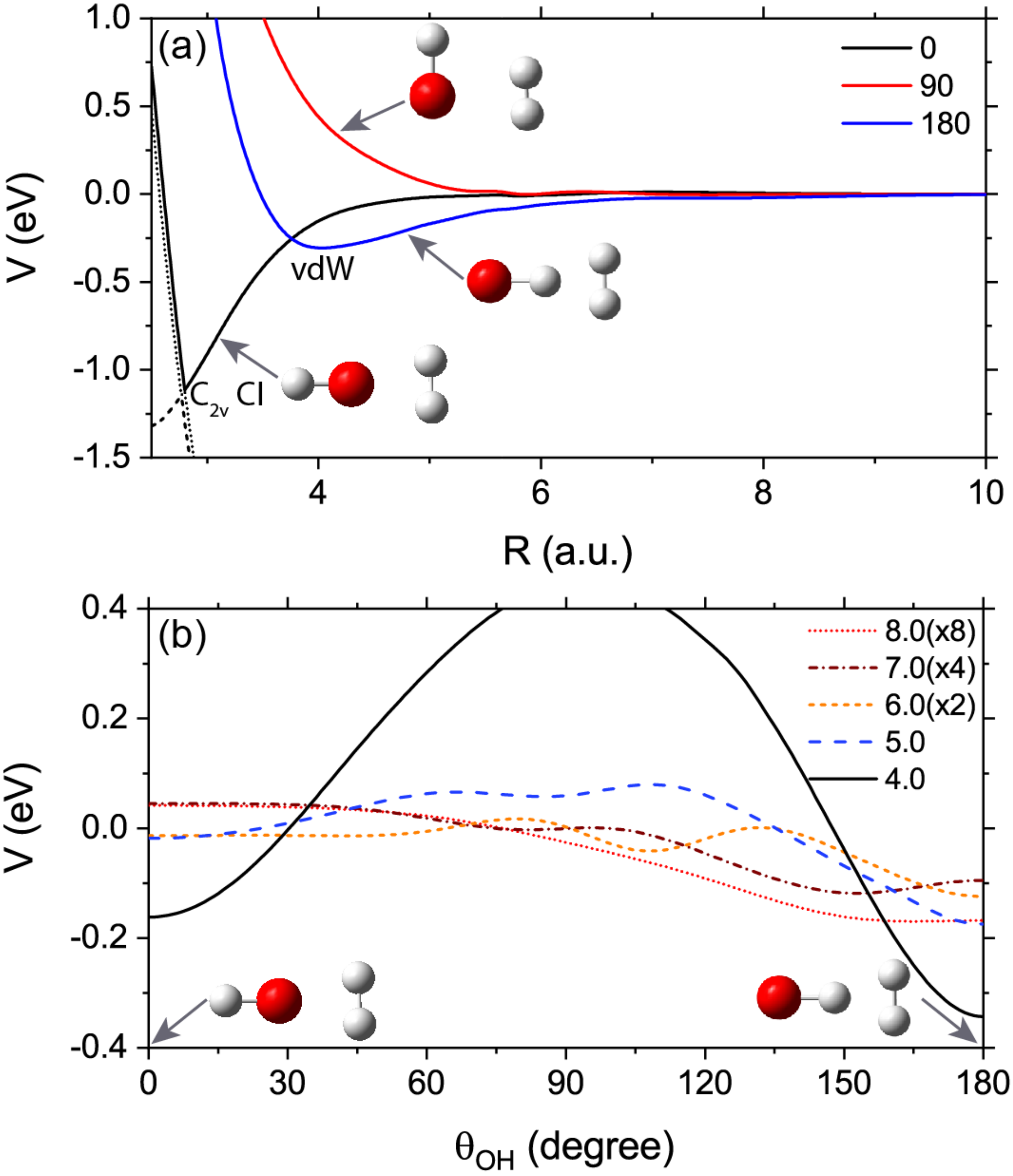

Supplementary Figure 1. One-dimensional plots of the adiabatic 3^2A state PES: (a) in the *R* coordinate for three OH orientations, i.e., θ_{OH} =0°, 90°, and 180°. The H-H and O-H bond lengths are fixed at the equilibrium values. The dashed and dotted lines show the C_{2v} CI. (b) in the θ_{OH} coordinate at different *R* values. The potential is relaxed with respect to the H-H and O-H bond lengths. In both panels, θ_{H_2} and φ are fixed at 90° and 0, respectively,

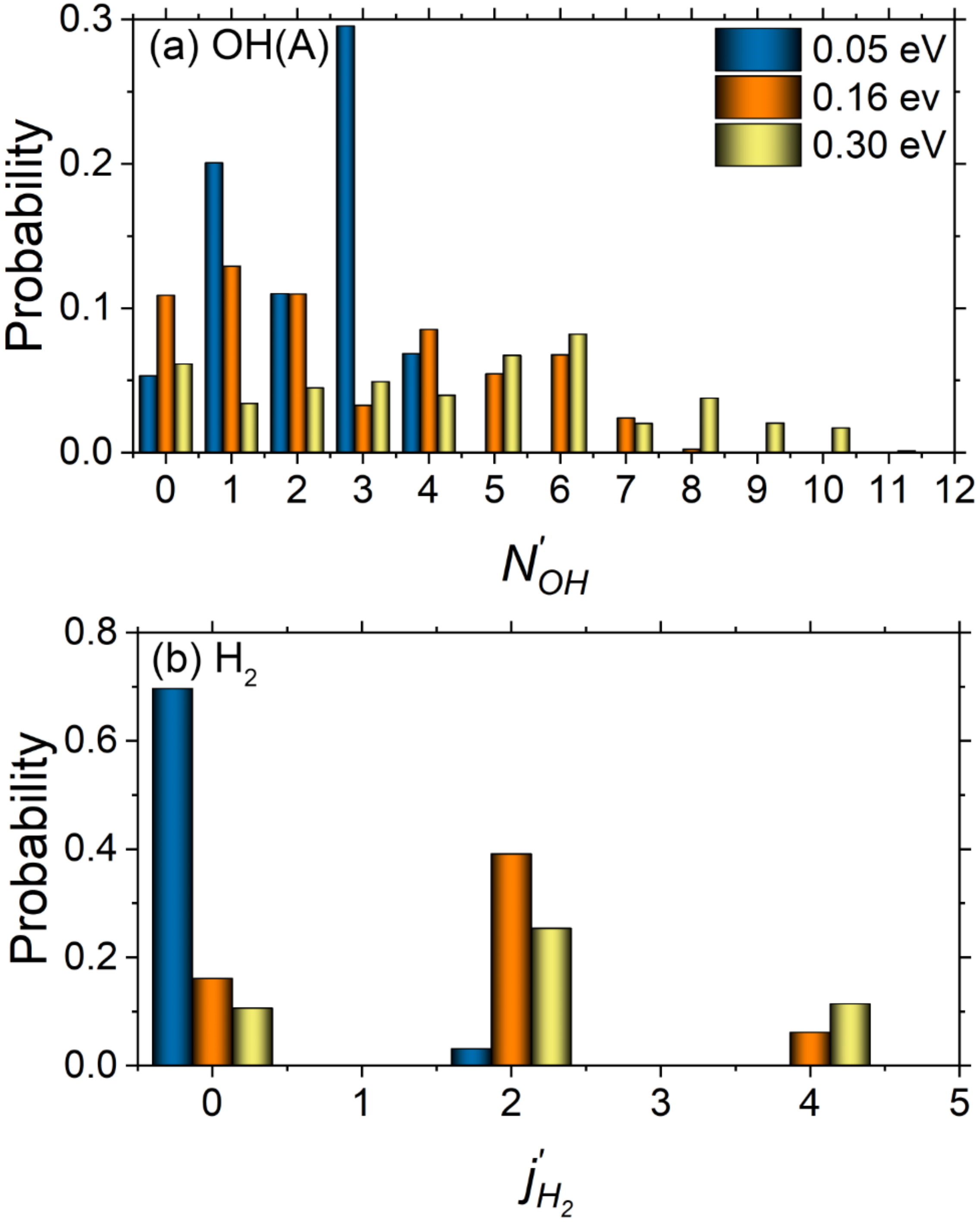

Supplementary Figure 2. OH(A) and H_2 rotational state-resolved distributions in the (in)elastic channel at three collision energies. Neither OH(A) nor H_2 has vibrational excitation.

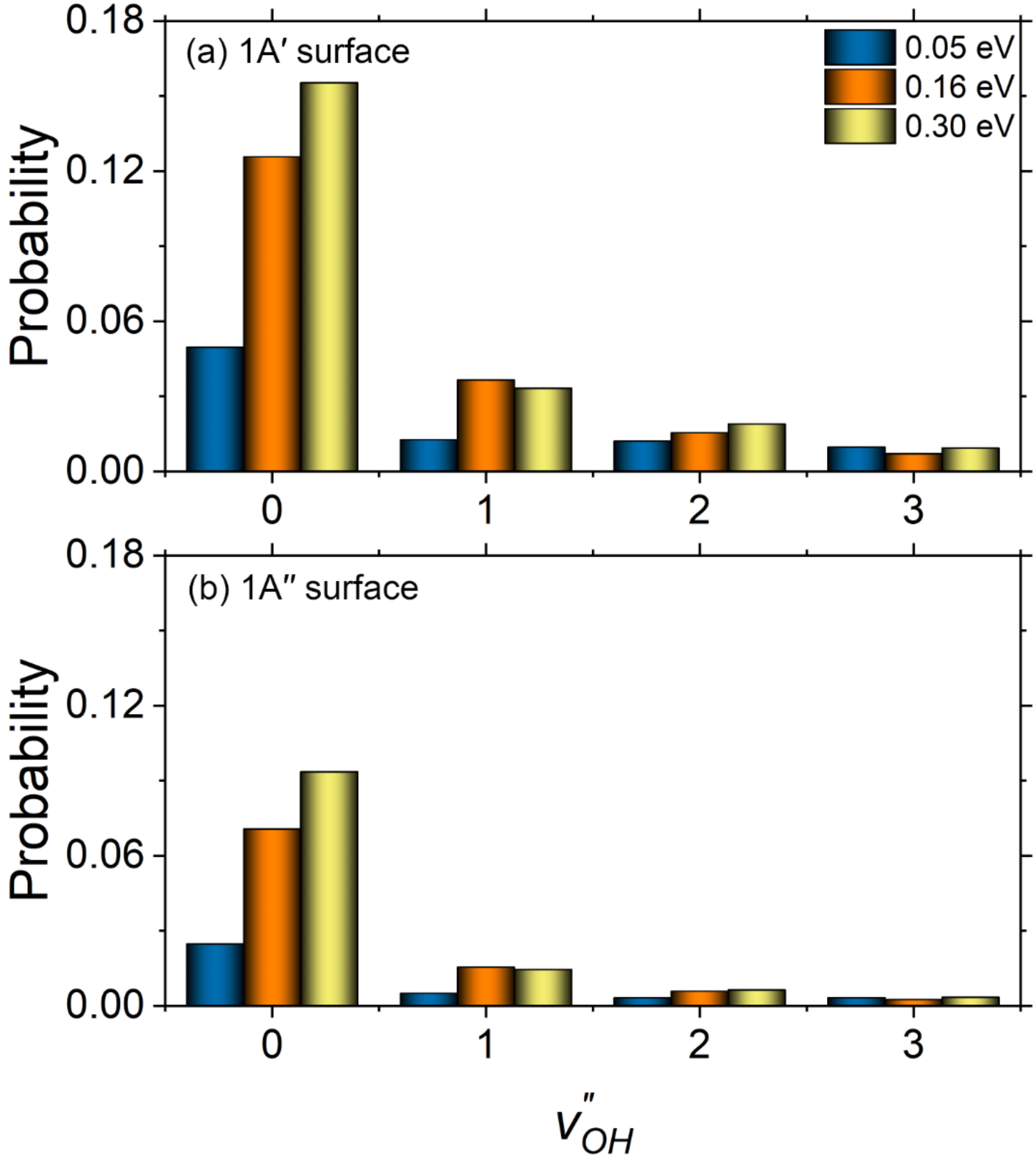

Supplementary Figure 3. Calculated OH(X) vibrational state-resolved probabilities at three collision energies. (a) on the 1A′ surface, (b) on the 1A″ surface.

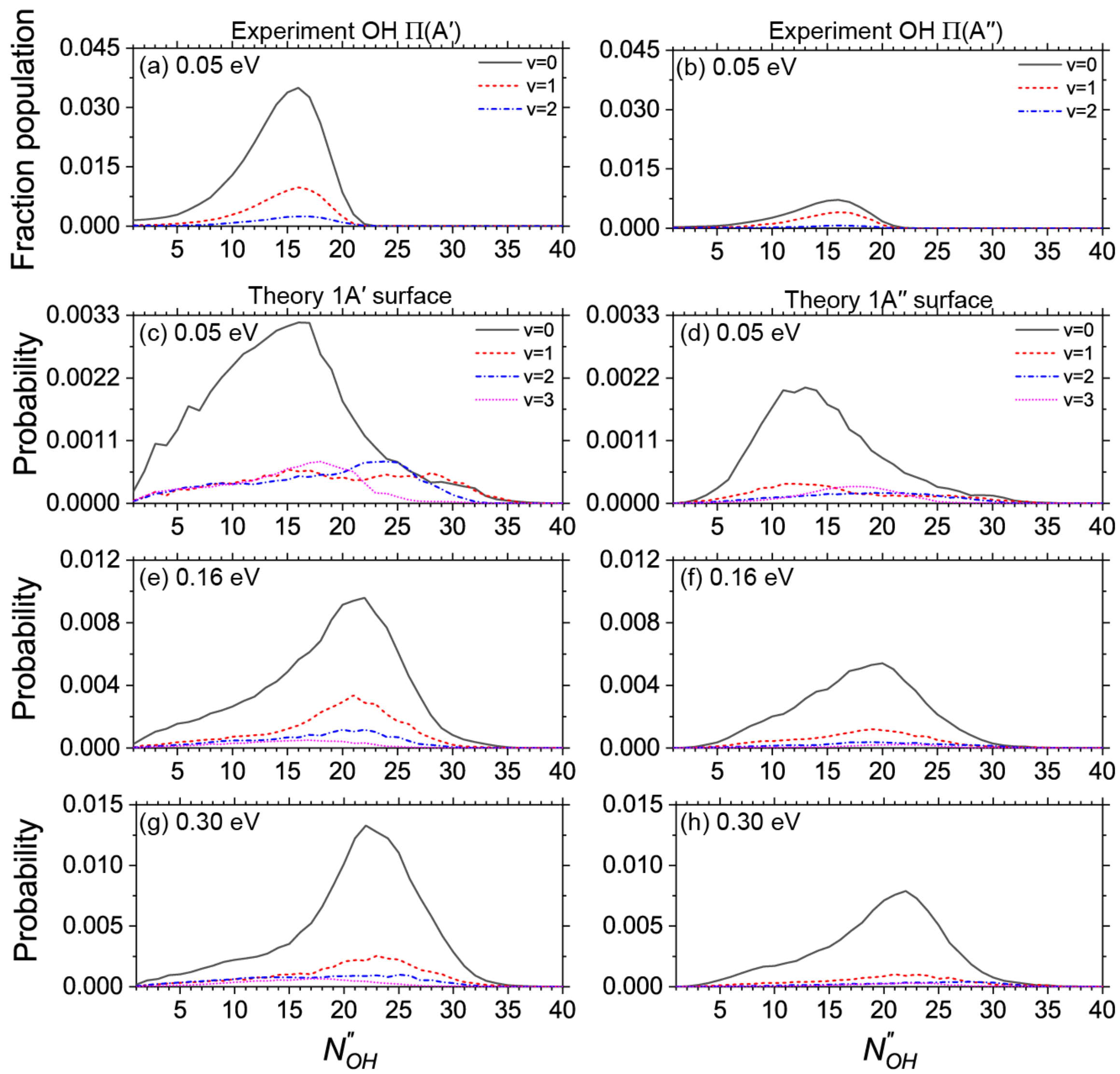

Supplementary Figure 4. The same as Figure 2 in the main text except that rotational states of each OH vibrational states are plotted separately. The experimental data shown here are the fitted functions of the raw data.[2]

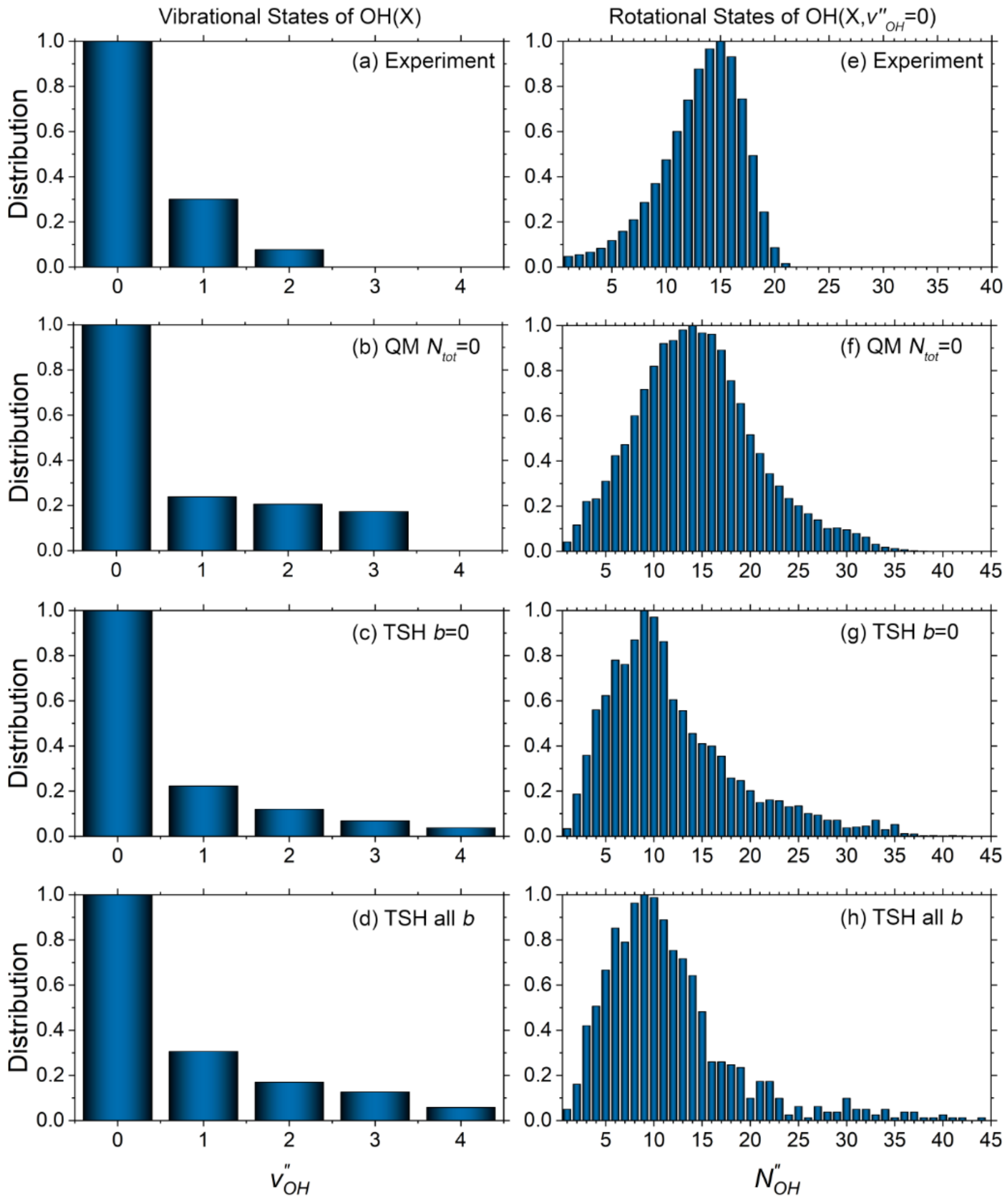

Supplementary Figure 5. Comparison of experimental, quantum, and TSH results for the OH(X) vibrational (left) and OH(X, v_{OH}=0) rotational (right) state distributions at the collision energy of 0.05 eV. (a) and (e): Fitted experimental results;[2] (b) and (f): Quantum mechanical results with N_{tot}=0; (c) and (g): TSH results with *b*=0; (d) and (h): TSH results including all relevant *b* values. The experimental results of the Λ-doublet are summed, and the theoretical results on the 1A′ and 1A″ surfaces are also summed.

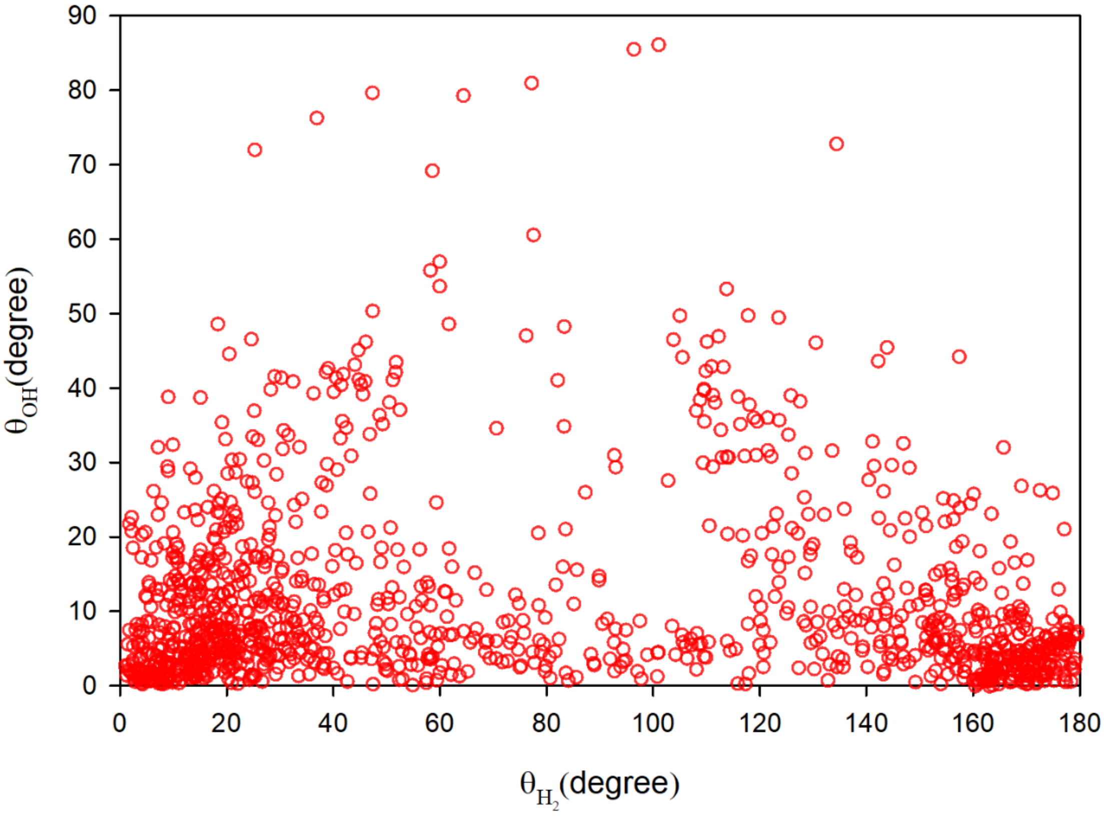

Supplementary Figure 6. Distribution of the hopping geometry for the 3^2A-2^2A transitions in the TSH calculations (*b*=0) at 0.05 eV.

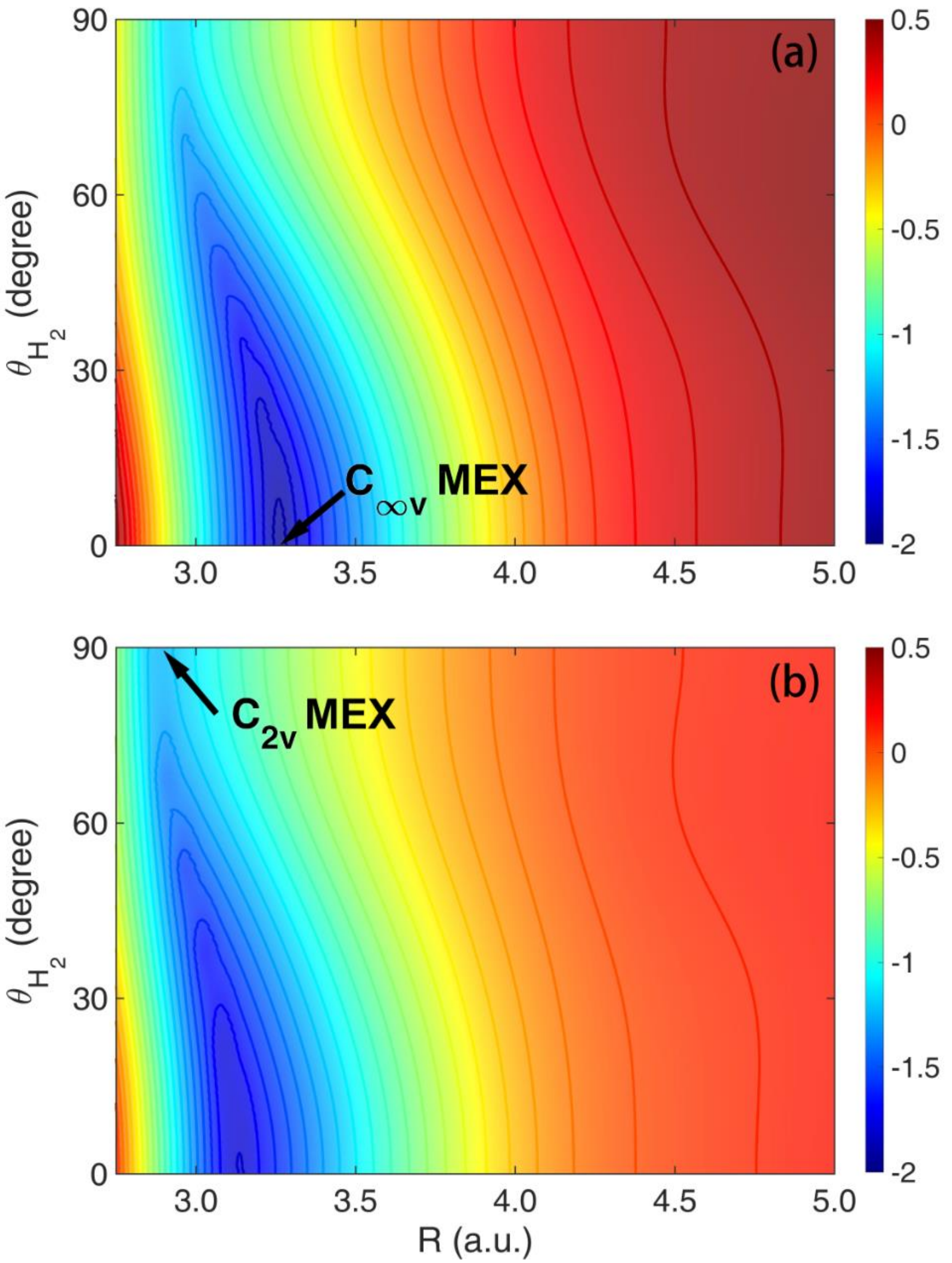

Supplementary Figure 7. Two-dimensional cuts of the 3^2A adiabatic surface along R and θ_{H_2}. The plots display the steep slope towards the linear $C_{\infty v}$ CI. The reference geometry is chosen at the $C_{\infty v}$ (a) and the 2^2A-3^2A(C_{2v}) (b) MEXs. The H-H and O-H bond lengths are fixed at their corresponding values, and θ_{OH} and φ are both 0 degree. (see Supplementary Table 1 for more details).

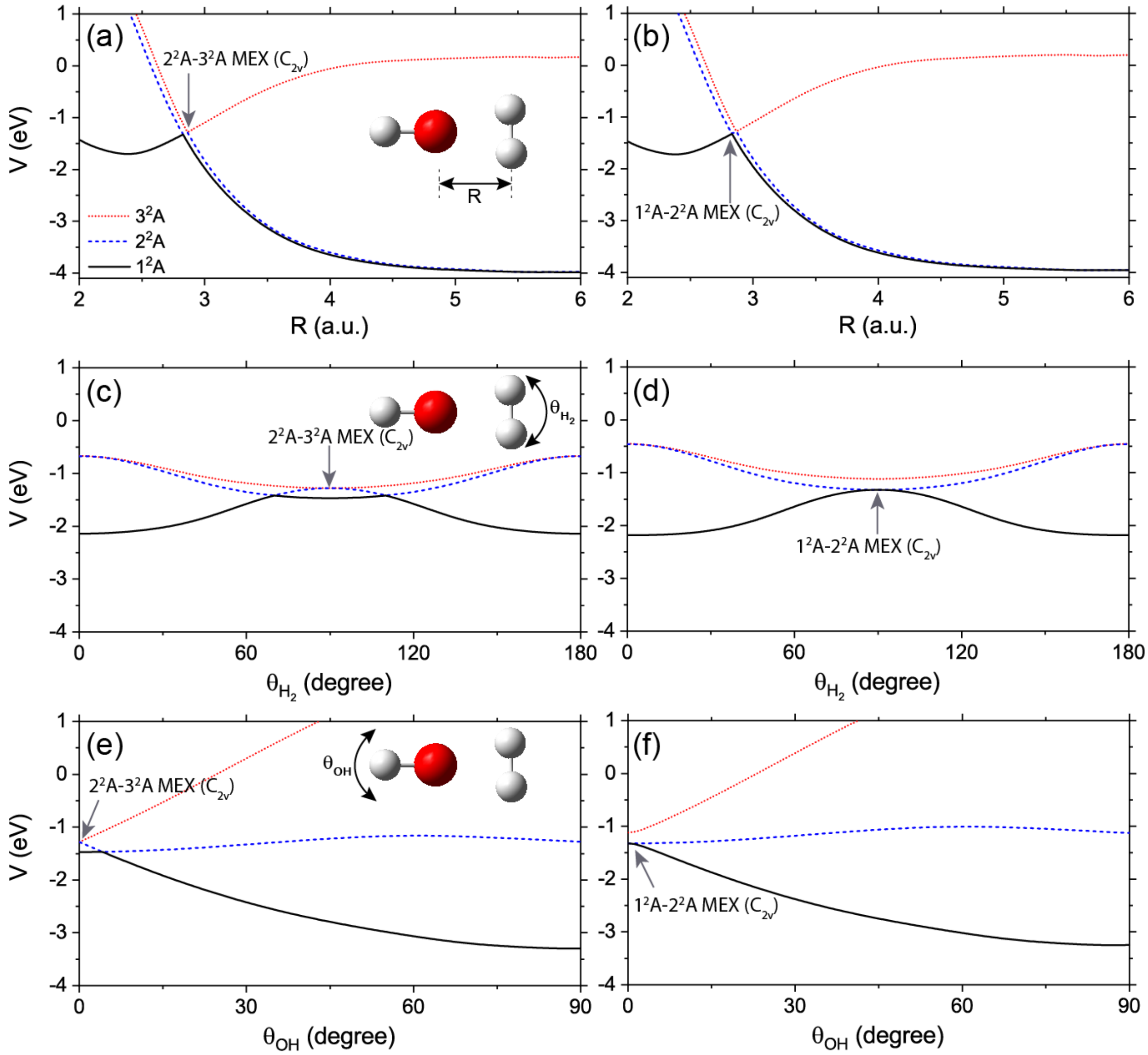

Supplementary Figure 8. One-dimensional cuts of the adiabatic surfaces. The same as Figure 5 of the main text except that the reference geometries are chosen at the 2^2A-3^2A(C_{2v}) and 1^2A-2^2A(C_{2v}) MEXs (see Supplementary Table 1).

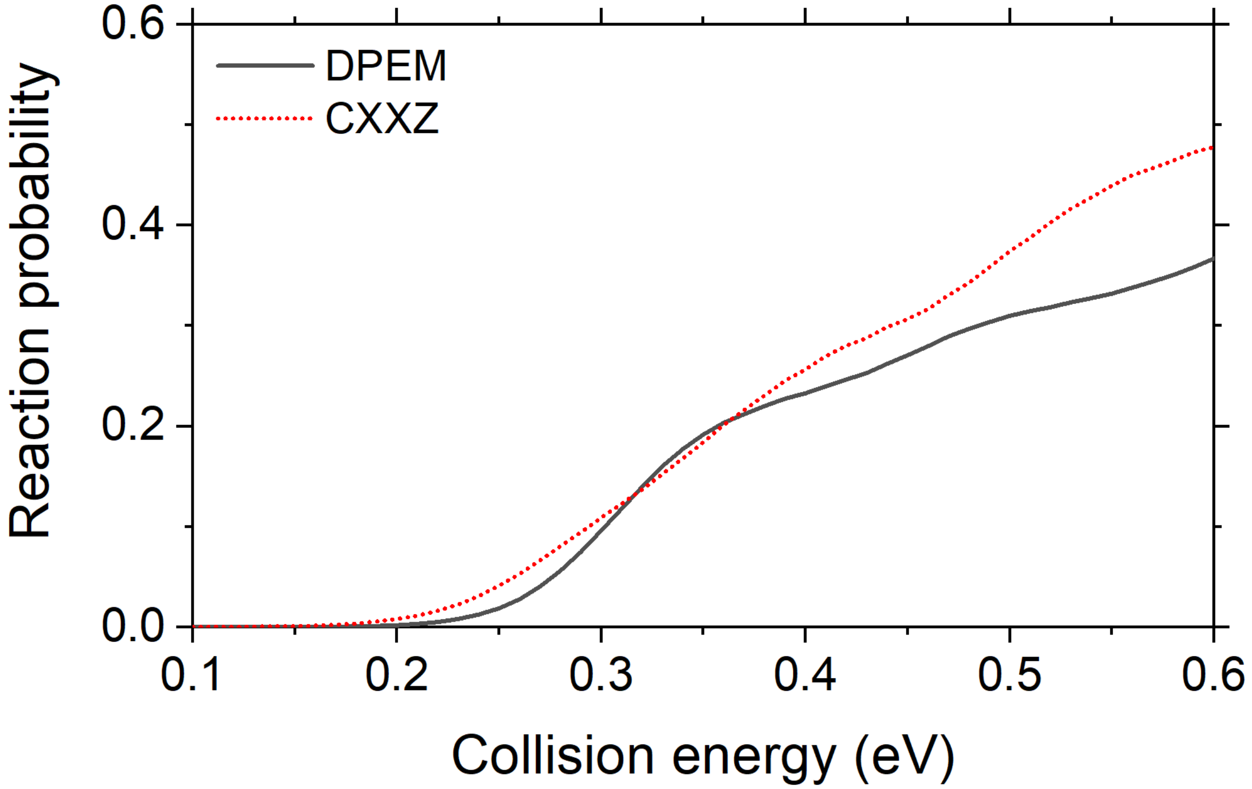

Supplementary Figure 9. Comparison of the reaction probability of the H_2+OH→H+H_2O reaction on the ground adiabatic state PES from the DPEM and the PES of Chen, Xu, Xu, and Zhang (CXXZ).[4] Both H_2 and OH reactants are in the ground ro-vibrational state.

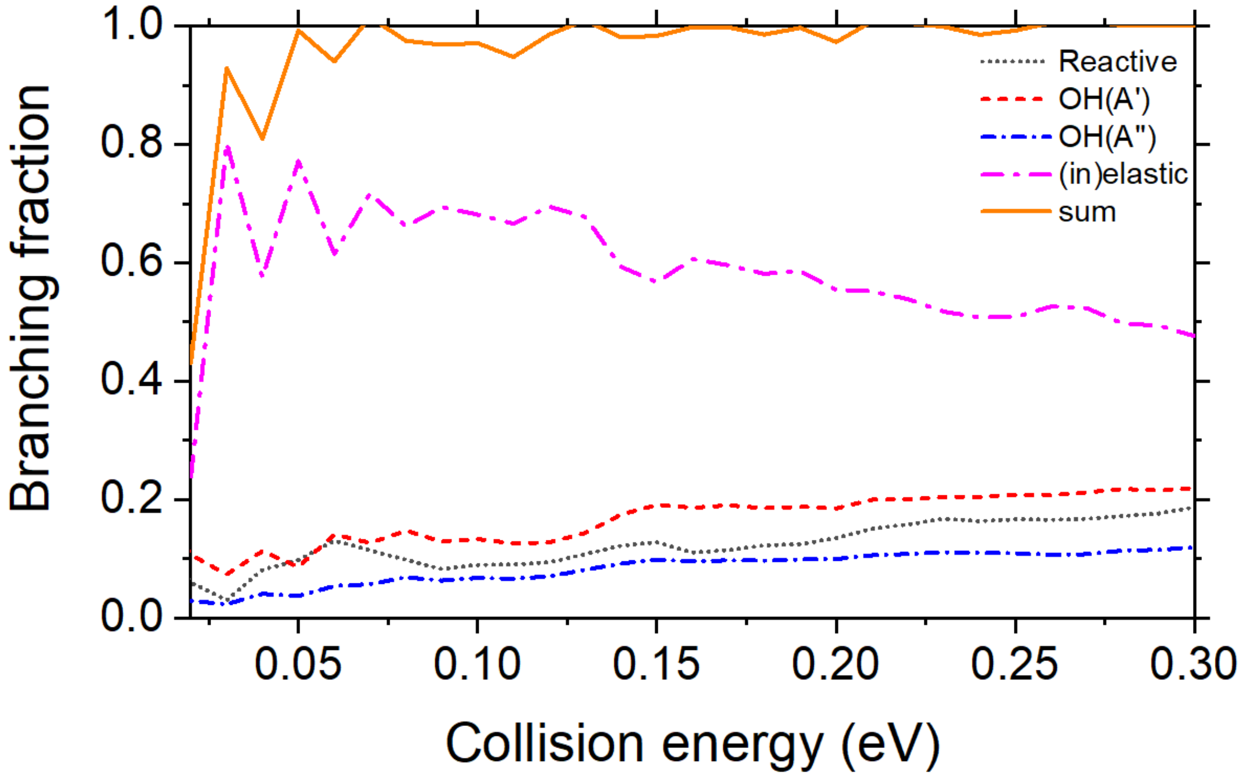

Supplementary Figure 10. Branching fractions of the reactive quenching, non-reactive quenching, and (in)elastic channels as functions of the collision energy. (Note: the (in)elastic channel populations might not be completely converged at low energies due to the long time trapping in the van der Waals well.)

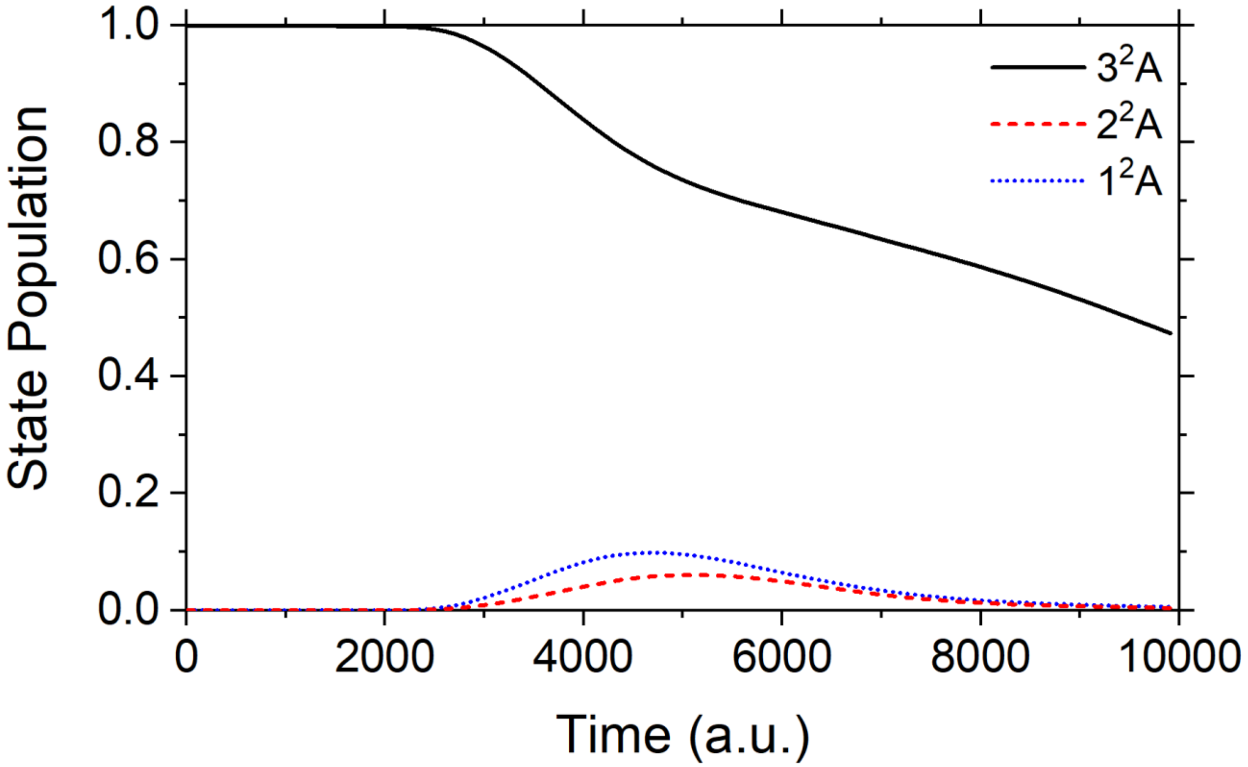

Supplementary Figure 11. Adiabatic state populations of the 1^2A, 2^2A, and 3^2A state wave packets as a function of time. The final decay after 5000 au is due to the absorbing potentials.

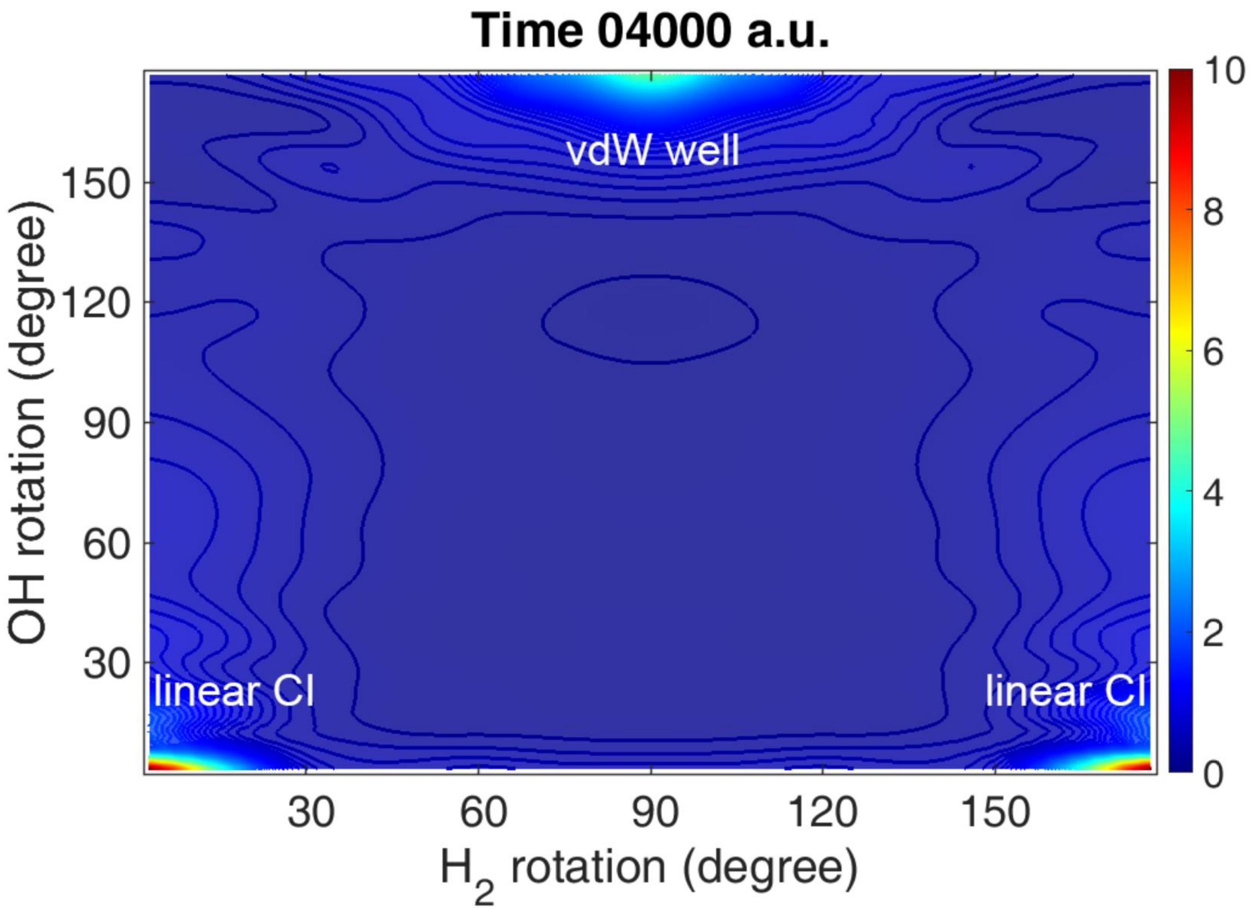

Supplementary Figure 12. 2D plot of the probability density of the wave packet on the excited state PES in the OH($A^2\Sigma^+$)+OH channel - a snapshot of the Supplementary Video 3 at the propagation time of 4000 a.u.
